# Multifunctional Nano-Contrast Agent Carriers: From Traditional Platforms to Next-Generation Theranostic Applications in Molecular Imaging

**DOI:** 10.3390/biomedicines14071552

**Published:** 2026-07-10

**Authors:** Danial Mirzaee, Marzieh Ramezani Farani, Maryam Ghasemzaei, Amir Gholami, Mohammad Seyedhamzeh, Iraj Alipourfard, Majid Farsadrooh, Mostafa Saffari, Mehdi Mirzaei, Omid Akhavan, Seyed Majid Ghoreishian, Yun Suk Huh, H. Bryan Riley, Mehdi Shafiee Ardestani

**Affiliations:** 1 Department of Biology, Faculty of Science, University of Tehran, Tehran 14155-6455, Iran; danialmirzaee@ut.ac.ir (D.M.); m.qassemzaei@gmail.com (M.G.); 2 NanoBio High-Tech Materials Research Center, Department of Biological Sciences and Bioengineering, Inha University, 100 Inha-ro, Incheon 22212, Republic of Korea; farani.marzi@inha.ac.kr (M.R.F.); yunsuk.huh@inha.ac.kr (Y.S.H.); 3 Faculty of Medicine, Kurdistan University of Medical Science, Sanandaj 66174-13391, Iran; amirgholamimd@gmail.com; 4 Zanjan Pharmaceutical Nanotechnology Research Center (ZPNRC), Department of Pharmaceutical Nanotechnology, School of Pharmacy, Zanjan University of Medical Sciences, Zanjan 45139-56184, Iran; m.s.hamzeh@gmail.com; 5 Department of Pharmacotherapy and Pharmaceutical Care, Faculty of Pharmacy, Medical University of Warsaw, 02-097 Warsaw, Poland; iraj.alipourfard@wum.edu.pl; 6 Renewable Energies Research Laboratory, Department of Chemistry, Faculty of Science, University of Sistan and Baluchestan, Zahedan 98167-45845, Iran; farsadroohmajid@gmail.com; 7 Department of Pharmaceutics, Faculty of Pharmacy and Pharmaceutical Sciences, Tehran Medical Sciences, Islamic Azad University, Tehran 19168-93813, Iran; mostafa.saffari@gmail.com; 8 Cancer Research Center, Shahid Beheshti University of Medical Sciences and Health Services, Tehran 19857-17443, Iran; mehdimirzaei4654@yahoo.com; 9 Department of Physics, Sharif University of Technology, Tehran 14588-89694, Iran; oakhavan@sharif.edu; 10 The Center for Energy and Environmental Solutions (CEES), College of STEM-T, South Carolina State University, Orangeburg, SC 29117, USA; 11 Department of Radiopharmacy, Faculty of Pharmacy, Tehran University of Medical Sciences, Tehran 14167-53955, Iran

**Keywords:** multifunctional nano-contrast agents, molecular imaging, theranostics nanomedicine, multimodal imaging, targeted nanocarriers, precision medicine, clinical translation

## Abstract

Multifunctional nano-contrast agent carriers are redefining molecular imaging by combining high-fidelity visualization with targeted delivery, controlled release, and, increasingly, therapeutic action. This review encompasses the development of nano-contrast platforms from conventional dendrimer, liposome, chitosan, and silica systems to modular nano-contrast platforms for multimodal, multi-parametric, and activatable imaging in clinically relevant environments. We dissect engineering strategies that govern surface chemistry, ligand organization, stimulus responsiveness, and microenvironmental sensing, and relate them to theranostic performance, immune system engagement, and quantitative image readouts. Biodistribution, pharmacokinetics, and safety are discussed from both classical and model-informed perspectives, with design principles that favor predictable behavior, manufacturability, and regulatory acceptance. Current clinical translation, regulatory pathway evolution, and market dynamics are critically reviewed to elucidate that a few nano-contrast agents have reached patients despite a widespread experimental landscape. Finally, we discuss emerging trends, including biomimetic and ultrasmall carriers, metal–organic and hybrid frameworks, AI-assisted design, digital twins, and precision medicine workflows, which are likely to shape the next-generation nano-contrast theranostics. By systematically relating material selection and carrier architecture to imaging function and translational limitations, this review suggests concrete research priorities for taking nano-contrast agents from sophisticated prototypes to robust, patient-tailored tools.

## 1. Introduction

Molecular imaging underpins modern diagnostics and treatment planning, enabling noninvasive visualization of molecular and cellular processes in vivo, often long before anatomical alterations are detectable [1,2,3,4]. Conventional small-molecule contrast agents, despite being clinically established, have limitations, including short circulation times, poor specificity, and narrow signal-generating capabilities, which reduce sensitivity and limit the ability to perform longitudinal monitoring [1,2,4]. Nanomaterial-based contrast agents have become a versatile alternative and can be designed to tailor pharmacokinetics, increase signal per unit dose, and provide additional biological functionality [1,2,3]. These nano-contrast systems comprise a wide range of compositions, including polymeric, lipidic, inorganic, and hybrid structures. They are now being explored for all primary imaging modalities (MRI, CT, PET/SPECT, optical, ultrasound, and photoacoustic imaging) [2,3,4]. In parallel, the growing clinical focus on precision medicine and real-time evaluation of response has led to strong demand for probes with high sensitivity and quantitative, reproducible readouts of biological responses [3,5]. Within this context, multifunctional nano-contrast agent carriers have been a central focus as next-generation tools for both diagnosis and image-guided therapy [1,2,3,4].

Beyond simple payload encapsulation, nanocarriers provide a modular framework for their design, enabling fine control over physicochemical parameters and biological interactions [1,2,3]. Size, shape, surface charge, and corona composition can be controlled to modulate circulation half-life, tissue extravasation, and cellular uptake. At the same time, high surface-area-to-volume ratios allow dense loading of contrast moieties, targeting ligands, and auxiliary functional groups [1,4,5,6]. This multivalency can increase receptor-mediated binding and local probe concentrations, thereby improving signal-to-noise ratio and enabling detection of low-abundance targets. In addition, nanocarriers have the advantage of incorporating multiple signal-generating elements, such as paramagnetic ions, high-Z atoms, radionuclides, or chromophores, within a single construct to enable multimodal or multiscale imaging strategies [1,2,3,5]. When combined with environment-responsive linkers or activatable reporters, nano-contrast systems can also provide dynamic information about microenvironmental cues, such as pH, redox status, or enzyme activity, opening avenues for functional and theranostic imaging that are not readily accessible with conventional small-molecule agents [3,5,6].

Historically, the evolution of nano-contrast carriers for molecular imaging has been guided by several archetypal platform families. Dendrimers, with their monodisperse, highly branched architecture and controllable generation number, have been widely investigated as scaffolds for magnetic resonance imaging (MRI), computed tomography (CT), positron emission tomography (PET), and hybrid imaging agents due to their interior cavities and surface groups, which allow for the co-loading of chelates, high-Z elements, and targeting ligands [7,8]. Liposomal systems provide biocompatible bilayer vesicles that can contain hydrophilic or lipophilic contrast agents, prolong blood residence time, facilitate passive accumulation in leaky vasculature, and enable flexible surface functionalization [1,4,5]. Polysaccharide-based carriers such as chitosan nanoparticles offer inherent biodegradability, mucoadhesion, and the ability to chelate metal ions or serve as hosts for hybrid magnetic or high-Z cores, supporting their use as both contrast agents and drug/gene delivery vehicles [5,9]. Silica-based nanoplatforms, in particular mesoporous and hybrid silica structures, offer robust, easily functionalized matrices that can co-localize dyes, metals, and drugs within well-defined porous architectures [4,10,11]. Together, these traditional platforms have provided the foundation for nano-contrast carrier engineering for molecular imaging [4,7,8,9,10,11].

More recently, there has been a shift in the field from single-function imaging probes to multifunctional theranostic nanoplatforms that combine diagnostic and therapeutic functions [3,5,12]. In such systems, classical contrast agents can be co-assembled with chemotherapeutics, radiosensitizers, photothermal or photodynamic agents, immunomodulators, or gene therapies for image-guided drug delivery, in vivo treatment monitoring, and adaptive treatment schemes [3,5,12,13]. Multimodal designs that combine, for example, MRI and PET, CT, or optical readouts further allow synergistic exploitation of complementary strengths in sensitivity, spatial resolution, and quantitative accuracy [1,4,12]. Nanocarriers also provide a convenient platform for incorporating stimuli-responsive features, such as pH-, redox-, enzyme-, or light-triggered release, thereby linking treatment to disease-associated microenvironmental cues and making treatment more spatially and temporally selective [3,10,13]. Collectively, these multifunctional constructs represent the concept of nanotheranostics, with the promise of more specific lesion characterization, improved patient selection, and tightly controlled, feedback-informed interventions in oncology and other fields [3,5,10,12,13].

Despite rapid conceptual advances, translating molecular imaging platforms based on nanocarrier contrast agents into clinically useful tools remains challenging. Complex architectures can pose challenges for large-scale, reproducible manufacturing, quality control, and long-term stability. At the same time, heterogeneity in size, surface chemistry, and aggregation state can result in unpredictable biodistribution and clearance [1,4,6]. Interactions with plasma proteins, immune cells, and biological barriers determine in vivo fate. They may cause off-target accumulation, immunogenicity, or altered pharmacokinetics, requiring careful optimization of surface coatings and stealth strategies [1,4,14]. Safety assessment is further complicated by the need to evaluate not only the inorganic or polymeric scaffold but also embedded metals, radionuclides, and therapeutics, including their degradation products and long-term retention in tissues [4,6,14,15]. Regulatory pathways for nanotheranostic agents are still evolving, and harmonized standards for characterization, preclinical evaluation, and clinical trial design are only starting to emerge. As a result, only a small percentage of the many nano-contrast systems reported in preclinical studies have progressed to early-phase clinical imaging or theranostic studies [1,4,6,12,14,15].

Existing reviews have presented valuable but often fragmented perspectives on nano-enabled imaging, typically focusing on a single material class, imaging modality, or therapeutic mechanism. Comprehensive analyses are available for dendrimer-based imaging and theranostic platforms, polysaccharide and chitosan nanoparticles, silica-based multimodal probes, and radionuclide-centered nanotheranostics in nuclear medicine [4,7,8,9,10,11,12]. However, these works rarely compare performance, design constraints, and translational bottlenecks between different families of nanocarriers within a specific context, namely as contrast-agent carriers whose main role is to orchestrate the loading, protection, targeting, and controlled release of diverse imaging and therapeutic payloads [1,3,4,6]. Furthermore, the systematic incorporation of new themes, such as sophisticated surface engineering, multi-layered targeting approaches, and interactions with the immune system, pharmacokinetic modeling, and regulatory evolution, remains limited [1,4,5,12]. There is therefore a need for a cross-cutting synthesis that bridges traditional carrier platforms and next-generation theranostic architectures, anchoring material and design discussions in clinically relevant metrics of imaging performance, safety, and translational feasibility [3,4,6,7,8,9,10,11,12].

**Figure 1:**
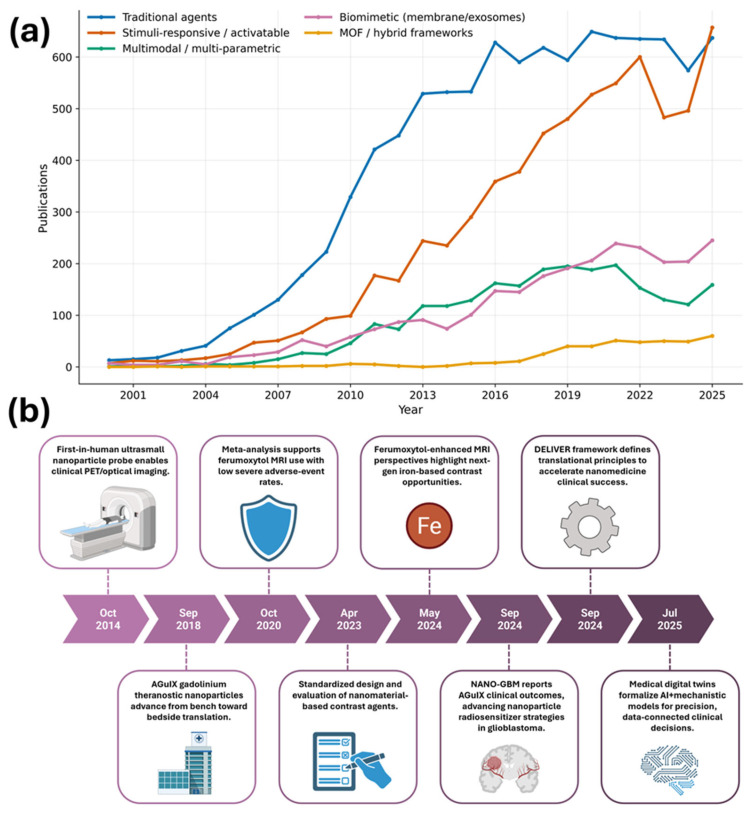
Publication growth and translational maturation of multifunctional nano-contrast agent carriers. (**a**) OpenAlex-derived annual publication counts (2000–2025) for major nano-contrast carrier themes, showing sustained activity in traditional agents alongside rapid growth in stimuli-responsive/activatable, multimodal/multi-parametric, and biomimetic (membrane/exosome) designs, with emerging contributions from MOF/hybrid frameworks. (**b**) Selected milestones illustrating the field’s progression from first-in-human ultrasmall nanoparticle imaging probes through increasing clinical evidence, standardization efforts, translational frameworks, and the recent emergence of medical digital twin concepts relevant to nano-contrast theranostics. Based on data from [1,16,17,18,19,20,21,22]. Panel (b) partially created in BioRender. Huh, Y. S. (2026). https://BioRender.com/xu1xpoh, accessed on 20 June 2026.

OpenAlex publication trends highlight the rapid expansion of research on nanocarrier contrast agents. At the same time, key clinical and translational milestones underscore the field’s shift toward clinically anchored, design-for-translation architectures (Figure 1a,b). In this review, we take a carrier-centered approach to bridge materials design, biological performance, and translational limitations of multifunctional nano-contrast agents. Following a brief conceptual background, Section 2 provides an overview of traditional platforms for molecular imaging, including dendrimers, liposomes, chitosan, and silica, and emphasizes how their physicochemical properties and clinical experience motivate the development of more advanced architectures. Section 3 then explores engineering strategies to make these systems multifunctional and targeted, such as surface and ligand chemistries, stimuli-responsive and smart designs, and multimodal or multi-responsive systems. Section 4 is devoted to theranostic biological applications, with particular attention to multimodal and multiparametric imaging and to interactions with the immune system. Section 5 presents an analysis of biodistribution, pharmacokinetics, and safety from both mechanistic and modeling perspectives. Section 6 examines the current clinical status, the evolution of regulatory pathways, and market translation, while Section 7 focuses on emerging trends, including biomimetic carriers, digitalization, and the integration of precision medicine. The article concludes in Section 8 with a synthesis of cross-cutting design principles and research priorities to accelerate the safe and effective clinical deployment of nano-contrast theranostics.

## 2. Traditional Nano-Carrier Platforms for Molecular Imaging

**Figure 2:**
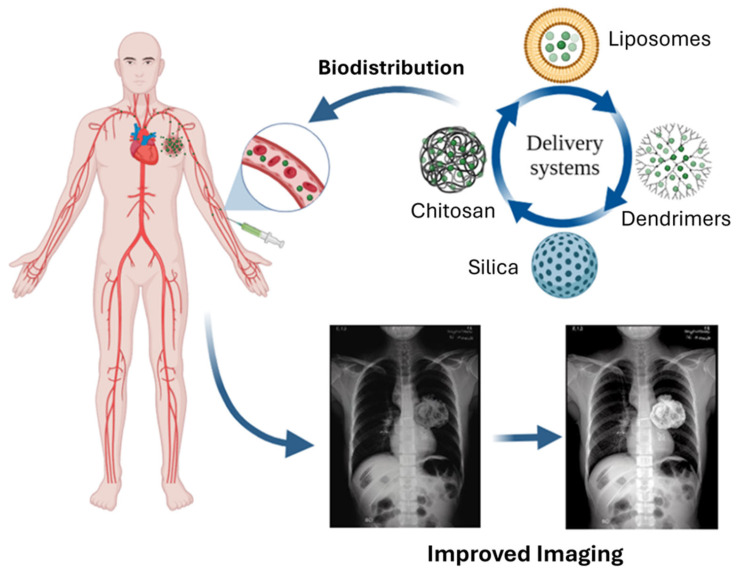
Traditional nanocarrier delivery systems improve contrast-agent biodistribution and imaging conspicuity. Partially created in BioRender. Huh, Y. S. (2026). https://BioRender.com/iqkowec, accessed on 20 June 2026.

Traditional nano-carrier platforms were developed to enhance the circulation time, bioavailability, and tissue specificity of small-molecule contrast agents, and they continue to serve as the architectural foundation for many next-generation theranostic systems. Dendrimers, liposomes, polysaccharide-based vehicles such as chitosan, and inorganic silica nanoparticles all provide engineered control over size, surface chemistry, and payload loading, allowing a higher contrast payload per particle and opportunities for multimodal imaging compared with conventional small-molecule iodinated or gadolinium (Gd) chelates [1,2,22,23]. Early efforts aimed to use these nanostructures as stealth depots for clinically used agents to modulate pharmacokinetics and minimize off-target toxicity, whereas subsequent designs leveraged multivalent targeting and co-loading of drugs and imaging reporters [1,2]. Although each platform has unique advantages, historical drawbacks in long-term safety, scalability, and batch-to-batch reproducibility motivate the evolution toward more sophisticated, multifunctional carriers, as considered in later sections [2,22,23]. Figure 2 provides an overview of these platforms. Additionally, a comparative overview of the main traditional carrier families (dendrimer-, liposome-, chitosan-, and silica-based systems) is given in Table 1. This comparison motivates the platform-specific design choices discussed in Section 2.1, Section 2.2, Section 2.3 and Section 2.4.

### 2.1. Dendrimer-Based Carriers

Dendrimers are monodisperse, hyperbranched macromolecules whose growth in generations produces well-defined architectures with many terminal functional groups and internal cavities, making them ideal scaffolds for high-density loading of contrast moieties and targeting ligands [24,39,40]. Poly(amidoamine) (PAMAM) and poly(propylene imine) dendrimers have been most widely explored in molecular imaging applications, in which peripheral amines or other functionalities can be derivatized with chelators for Gd(III), manganese, or other radiometals, as well as fluorophores or radionuclide-bearing ligands [22,41]. Compared with small-molecule agents, dendrimer-based formulations have increased relaxivity per molecule, prolonged intravascular residence, and tunable hydrodynamic diameters that can provide either blood-pool imaging or enhanced permeation and retention (EPR)-mediated tumor accumulation [24,39,40]. These structural attributes provide the basis for their early designation as model macromolecular contrast-agent carriers and have established a benchmark for subsequent polymeric and inorganic platforms.

Dendrimeric MRI contrast agents typically have high longitudinal relaxivities, obtained by clustering several Gd chelates at specific distances from the dendrimer surface, where restricted rotational correlation times enhance water–proton relaxation compared with freely tumbling chelates [39,40]. Generation-dependent studies using PAMAM–Gd complexes have revealed that increasing generation up to G5–G6 enhances r_1_ relaxivity and prolongs blood half-life, resulting in substantial vascular and hepatic enhancement in vivo [40]. Kojima et al. [39] showed that functionalization with polyethylene glycol (PEGylation) reduces nonspecific uptake by the reticuloendothelial system and prolongs circulation. However, excessive shielding may reduce water accessibility to Gd and thus relaxivity. These designs were, in part, in response to concerns about Gd retention and nephrogenic systemic fibrosis with some small-molecule agents, and focused on the ability of dendrimer carriers to modulate Gd pharmacokinetics and, potentially, toxicity when used in combination with stable chelates [22,42].

In addition to peripheral derivatization, the dendrimer architecture supports two modes of contrast generation. In the first, chelates of Gd(III) are covalently attached to surface groups that are located at the terminus of the macromolecules; the restricted rotational mobility of the macromolecule slows the rotational correlation time (τR) of the bound water–Gd vector, thereby increasing the relaxivity (r_1_) relative to free chelates [43,44]. In the second, the internal cavities and branching voids of the dendrimer host can encapsulate or entrap inorganic cores, including dendrimer-entrapped gold nanoparticles, which generate contrast through X-ray attenuation for CT [45]. Both modes are affected by the generation number: increasing the generation lengthens τR and raises r_1_ per Gd up to a saturation point, beyond which slow water exchange limits further relaxivity gains [43,44]; higher generation number also gives larger internal voids, which can template larger entrapped cores [45,46].

Beyond MRI, dendrimer scaffolds have been extended to multimodal and theranostic constructs where optical dyes, radionuclides, or magnetic nanoparticles are co-assembled with therapeutic payloads within a single architecture. Dendrimer–Gd systems have been used with fluorescent reporters for intraoperative imaging and with radiometal chelators for dual PET/MR or SPECT/MR imaging, taking advantage of the dendrimer’s multivalency to regulate the stoichiometry and spatial arrangement of each component [24,41]. More recently, small-molecule chemotherapeutics or photosensitizers have been incorporated into their design, facilitating image-guided drug delivery and photodynamic therapy while preserving diagnostic signal [1,41]. However, there are various challenges for clinical translation, including incomplete biodegradation of higher-generation dendrimers, complement activation, and complex manufacturing. Such immune liabilities strongly depend on the surface charge, because cationic, amine-terminated PAMAM dendrimers are more likely to induce complement activation and cytotoxicity, while neutralized surfaces resulting from PEGylation or acetylation markedly reduce these effects, enhancing biocompatibility [47,48,49,50]. Thus, terminal-group engineering is an important safety design parameter, along with generation control and payload loading, to move dendrimer-based imaging carriers from the bench to clinical trials [49,50].

### 2.2. Liposome-Based Contrast Agents

Liposomes, composed of phospholipid bilayers that encapsulate an aqueous core, are among the most advanced nanocarrier systems in clinical practice and have been extensively modified to deliver imaging agents and chemotherapeutics. Their structural versatility enables loading the core with hydrophilic contrast agents and the bilayer with hydrophobic or amphiphilic probes. Furthermore, PEGylation and controlled size (typically 80–150 nm) enable prolonged circulation and EPR-driven tumor accumulation. Liposomal encapsulation of iodinated agents, gadolinium, or high-Z metal nanoparticles has been used to increase blood-pool and organ-specific CT or MRI contrast, with the advantages of higher payloads, tunable pharmacokinetics, and, in some designs, triggered release [1,22,28]. In parallel, the long clinical history of liposomal drugs makes it easier to gain regulatory familiarity with the core formulation technologies, although imaging-specific products still need to meet different dosing and safety profiles than therapeutics [2,22,28].

Two main compartments within liposomes (aqueous and phospholipid bilayer) provide two different approaches to contrast generation. Amphiphilic Gd-chelating lipids, such as Gd-DTPA-bis(stearylamide) and other DOTA derivatives, can be incorporated directly into the bilayer, with their chelate head groups facing the aqueous interface, while hydrophilic Gd chelates or iodinated agents can be encapsulated in the aqueous core at high concentration. Relaxivity is also strongly affected by compartmentalization, as the rate of water exchange across the bilayer to the paramagnetic center differs markedly for core-encapsulated chelates and bilayer-anchored chelates, the latter of which will have a higher relaxivity, r_1_, due to easier water access [51,52]. In addition to paramagnetic and iodinated payloads, the aqueous milieu can also accommodate quantum dots, radionuclides in solution, or ultrasmall iron oxide cores, which allows the creation of multimodal constructs combining optical, nuclear, and magnetic readouts (magnetoliposomes) [28,53].

**Figure 3:**
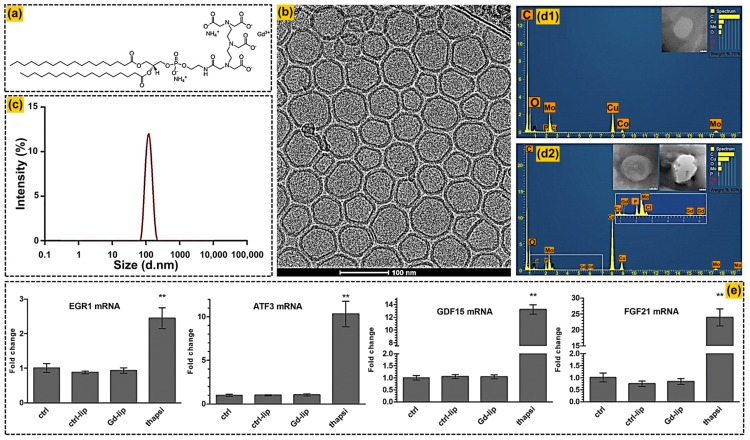
Physicochemical characterization and hepatocellular stress screening of Gd-chelating nanoliposomes (Gd–lip). (**a**) Chemical structure of the PE–DTPA(Gd) chelating lipid used to anchor Gd in the liposomal bilayer. (**b**) Cryo-TEM image confirming a monodisperse unilamellar vesicle morphology with enhanced membrane contrast consistent with Gd incorporation. (**c**) Hydrodynamic size distribution measured by DLS. (**d**) TEM–EDX spectra of control liposomes (**d_1_**) versus Gd–liposomes (**d_2_**), confirming the presence of Gd in the formulation. (**e**) RT–PCR analysis of early response/toxicity marker genes (*EGR1*, *ATF3*, *GDF15*, *FGF21*) in differentiated HepaRG cells after 24 h exposure to control liposomes or Gd–liposomes; thapsigargin (3 μM, 24 h) is shown as a positive stress control (mean ± s.d., n = 4; ** *p* < 0.01 vs. negative control). Adapted with permission from ref. [29]. Copyright 2020, Springer Nature.

Liposomal MRI contrast agents often involve gadolinium (Gd) chelating lipids, such as phosphoethanolamine-DTPA, being part of the lipid bilayer, or the aqueous encapsulation of hydrophilic chelates, where relaxivity is dependent on the membrane composition, packing, and rotational dynamics [28,29,54]. Šimečková et al. [29] reported negative surface charge, colloidal stability, and negligible cytotoxicity of Gd-containing nanoliposomes incorporating PE-DTPA and cholesterol in hepatocytes and macrophages, findings that support the use of nanoliposomes as MRI theranostic platforms for thrombolytic delivery (Figure 3). Aptamer-conjugated, Gd-loaded liposomes targeting tenascin-C-overexpressing tumor cells also show that the conjugation of the ligand can greatly improve the cellular uptake and T_1_ contrast compared to non-targeted formulations, while retaining the nanometric size and acceptable relaxivity [54]. Collectively, these studies demonstrate that liposomal composition and surface chemistry determine both imaging performance and biological response.

Liposomal carriers are also readily adapted to multimodal and theranostic constructs that combine MRI, CT, optical, or nuclear imaging with controlled drug delivery. Xia et al. [28] recently conducted a systematic review of liposome-based probes that combine Gd, iodinated compounds, radionuclides, and near-infrared dyes within the same vesicle and enable, for instance, dual MR/fluorescence or PET/CT imaging and image-guided surgery. In the formulation of Gd-liposomes, co-loading with thrombolytics or chemotherapeutics enables simultaneous visualization of thrombi or tumors and localized therapy, underscoring liposomes’ role as clinically relevant theranostic depots [28,29]. Aptamer- or antibody-decorated liposomes further enhance molecular specificity at the expense of added formulation complexity and potential immunogenicity [54]. Traditional liposomal imaging agents thus define a mature yet evolving platform in which trade-offs among payload, stability, targeting, and manufacturability continue to shape translational prospects.

### 2.3. Chitosan-Based Contrast Agents

Chitosan, a partially deacetylated derivative of chitin, is a cationic, mucoadhesive polysaccharide widely used in nanomedicine due to its biocompatibility, biodegradability, and abundant functional groups available for chemical modification and polyelectrolyte complex formation. Conventional preparations of chitosan nanoparticles are based on ionic gelation or polyelectrolyte complexation, yielding submicrometer-sized particles whose surface charge and hydrophilicity can be adjusted by the degree of deacetylation, molecular weight, and copolymers [9]. Recent reviews emphasize the versatility of these properties for chitosan as a scaffold for cancer diagnosis and therapy, in the form of a matrix or coating for magnetic, optical, or nuclear contrast agents, and as stimuli-responsive nanoplatforms [32]. In imaging applications, the colloidal stability of chitosan can be exploited, along with enhanced cellular internalization due to electrostatic interactions with negatively charged membranes and the possibility of pH- or redox-triggered release of co-loaded therapeutics, thereby positioning it as a step between classical polymeric carriers and more sophisticated theranostic constructs [9,32].

One important factor to consider for chitosan-based carriers is that the imaging signal is not generated by chitosan; rather, chitosan forms a biocompatible shell that stabilizes and functionalizes an inorganic core whose intrinsic properties produce the imaging signal. Most frequently used cores are superparamagnetic or ultrasmall superparamagnetic iron oxide (SPIO/USPIO) nanoparticles, whose superparamagnetism results in a signal void that serves as negative MRI contrast [55,56]. Alternatively, chitosan has been coated onto gold or mixed-metal ferrite cores for use in CT or combined magnetic applications [57,58]. Comparative studies show that the magnetic properties of the ferrite cores, namely, the transverse relaxivity and tunable saturation magnetization, depend on the ferrite composition, with chitosan-coated Co_0.5_Zn_0.5_Fe_2_O_4_ exhibiting tunable magnetic properties distinct from the conventional chitosan-coated magnetite (Fe_3_O_4_) core, which is the most extensively characterized reference for T2 contrast [55,58]. In both instances, the major influence on the colloidal stability, biocompatibility, and availability of functional groups for further modification is the chitosan shell.

A representative class of nano-contrast carriers based on chitosan comprises those with magnetic cores and chitosan or chitosan derivative shells, which provide both T2 MRI contrast and enhanced biocompatibility. As noted above, Worawong & Onreabroy prepared Co_0.5_Zn_0.5_Fe_2_O_4_ nanoparticles coated with chitosan that resulted in colloidally stable dispersions with superparamagnetism and transverse relaxivity as potential for contrast enhancement in MRI [58]. The chitosan shell provides the benefit of aqueous stability, the presence of reactive amine and hydroxyl groups for further functionalization, and possibly alleviates the toxicity of the ferrite core, while preserving nanoscale dimensions that favor vascular circulation and tissue penetration [9,58]. Broader surveys of nanosystems based on chitosan highlight their applications as platforms for multimodal imaging (e.g., MRI/fluorescence) and as immunomodulatory carriers that affect the tumor microenvironment, although most studies remain preclinical, and systematic examinations of long-term biodistribution and degradation products are limited [32].

### 2.4. Silica-Based Contrast Agents

Silica nanoparticles, and more specifically mesoporous and hollow mesoporous silica nanoparticles (MSNs and HMSNs), are prototypical nanocarriers from the family of inorganic materials, which are characterized by high surface area, adjustable pore size, strong framework chemistry, and flexibility in surface decoration [35,59,60]. These characteristics enable independent control of the core composition and of the thickness and porosity of the shells, allowing the co-loading of diagnostic and therapeutic agents in physically segregated domains. Mesoporous silica platforms have been widely explored for drug delivery and molecular imaging, owing to the ability to dope or decorate the siloxane network with paramagnetic ions, high-Z metals, fluorophores, and radionuclides without compromising structural integrity [35]. Hollow or rattle-type architectures further increase internal volume, reduce density, and provide additional room for payloads and, in some designs, for gas or perfluorocarbon filling for ultrasound contrast [60]. These features make the silica-based carriers a canonical traditional inorganic nanoplatform from which many current theranostic designs have been developed.

Silica-based carriers may be further divided by their internal architecture, which determines the payload strategy and contrast mechanism. Solid silica nanoparticles lack internal porosity, so dyes, paramagnetic ions, or other reporters must be incorporated directly into the dense siloxane network during synthesis, either noncovalently by entrapment or covalently through the use of organoalkoxysilane precursors [61]. In contrast, mesoporous silica nanoparticles contain ordered cylindrical pore channels with a large internal surface area for adsorptive loading of drugs and contrast agents, while retaining the framework for surface functionalization [61,62]. A third architecture type is hollow or rattle-type shells, which have a relatively large volume of internal space, which may be filled with a smaller core particle in a yolk–shell configuration, or filled with gas or perfluorocarbon for ultrasound contrast [63]. The siloxane framework can be doped with paramagnetic centers (for example, Gd(III) or Mn(II/III) ions) or modified with organic bridging groups that can adjust the hydrophilicity and degradation rate [61,62].

Concrete implementations of silica-based nanocarrier contrast agents are used across many imaging modalities. Chu et al. [59] developed mesoporous silica nanoparticles coated with microwave-synthesized naked platinum nanoparticles (MSNs-Pt) and conjugated with a near-infrared dye to obtain a dual CT/optical imaging agent with a hydrodynamic diameter of ~50 nm and a high Pt loading capacity that substantially enhances X-ray attenuation and in vivo tumor contrast. Separately, a functionalized silica nanoparticle platform with chelated gadolinium and organic fluorophores in a mesoporous matrix has been reported as a bimodal MRI/optical contrast agent, demonstrating strong T_1_ MRI signal enhancement and high fluorescence with controllable surface charge and hydrodynamic size [64]. Hollow mesoporous silica nanoshells filled with perfluorocarbon or bearing magnetic cores have also enabled MR/US-guided drug delivery, highlighting the versatility of silica as a carrier for multimodal imaging [60,64].

Despite their versatility, traditional silica-based platforms raise important safety and translational questions. Mesoporous silica nanoparticles may have relatively large hydrodynamic diameters and high aspect ratios, which may favor uptake by macrophages in the liver and spleen, raising concerns about long-term retention and organ-specific toxicity if the degradation process is slow. Xu et al. [35] underline that biodegradation kinetics are strongly related to particle size, porosity, and surface chemistry, with smaller-sized, thin-shelled, or organically modified particles biodegrading more quickly to excretable silicic acid, whereas dense or oversized constructs may persist for longer. These observations are reminiscent of broader discussions of nanomaterial-based contrast agents, in which achieving high contrast payloads and multimodality, with predictable clearance and low chronic accumulation, are central design challenges [1,23]. As a result, recently developed silica-based theranostics have increasingly been integrated with biodegradable frameworks, responsive linkages, and surface stealth to overcome the limitations of traditional formulations.

## 3. Engineering Multifunctional and Targeted Nano-Contrast Carriers

The design focus in this section is less on selecting a carrier scaffold, as described in Section 2, and more on engineering its outer and inner architecture to control biological fate and signal generation and action in a therapeutic, integrated manner [65]. Building on the platforms of dendrimers, liposomes, chitosan, and silica, sophisticated nano-contrast carriers take advantage of carefully tuned surface chemistries, hierarchical ligand patterns, and internal functional domains to couple imaging readouts with microenvironment sensing and therapy [32,65,66,67,68,69]. At the same time, more and more targeted motifs and stimuli-responsive elements are selected with specific clinical questions in mind, such as margin delineation, early response assessment, or immune monitoring in defined patient subgroups [65,67,70]. The following subsections, therefore, focus on strategies to convert such traditional nanocarriers into programmable theranostic delivery systems, first through rational surface and ligand engineering, and subsequently by incorporating smart responsiveness and by combining multimodal signaling and personalized targeting concepts with knowledge of disease biology and emerging precision medicine frameworks. Table 2 summarizes the main engineering handles available for nano-contrast carriers and correlates each design option with its dominant biological consequences (e.g., corona formation, immunological recognition, uptake pathways) and imaging results (e.g., specificity, signal activation, quantitative interpretability).

### 3.1. Advanced Surface Chemistries and Ligand Engineering

**Figure 4:**
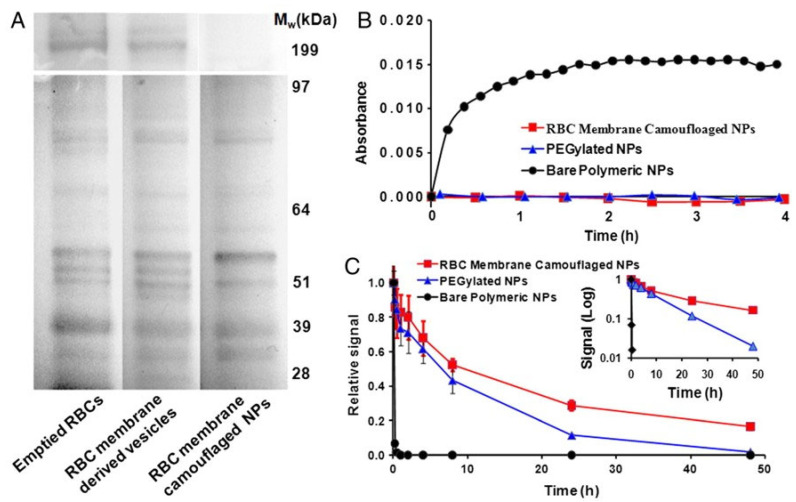
Biomimetic erythrocyte-membrane camouflage enhances nanoparticle “stealth” behavior. (**A**) SDS-PAGE comparison indicating retention of erythrocyte membrane proteins after coating polymeric nanoparticles. (**B**) Serum stability assay (absorbance at 560 nm over time) comparing RBC-membrane-camouflaged nanoparticles with PEGylated and bare polymeric controls. (**C**) In vivo circulation kinetics showing prolonged persistence of RBC-membrane-coated nanoparticles versus PEGylated or uncoated controls. Reprinted with permission from ref. [106]. Copyright 2011, National Academy of Sciences.

Surface chemistry is the major interface between nano-contrast carriers and the biological environment, determining the composition of the protein corona, colloidal stability, immunologic recognition, and target binding. Contemporary approaches go beyond simple PEGylation and have introduced mixed-ligand shells that combine stealth polymers, zwitterionic or biomimetic coatings, and reactive handles for conjugating targeting ligands and therapeutics. Systematic investigations of the surface functionalization of inorganic nanoparticles indicate that the mode of ligand binding, packing density, and chain flexibility are important factors controlling not only colloidal stability but also water accessibility and relaxivity, which are pertinent to MRI and multimodal readouts [103,104,105]. Biomimetic cell membrane coatings offer a compelling alternative to purely synthetic stealth polymers: erythrocyte-membrane-camouflaged nanoparticles retain native membrane proteins, remain stable in serum, and prolong circulation compared with PEGylated or uncoated controls (Figure 4) [106]. Reviews of surface-functionalized nanoparticles also emphasize the importance of properly selecting anchoring groups (thiols, silanes, phosphonates, catechols) and spacer architectures to reduce toxicity, reduce nonspecific uptake, and increase cellular internalization efficiency [104,105]. Within the imaging context, these design principles are now applied to classical contrast-carrier cores such as iron oxides, silica, and high-Z metals, enabling stable, high-payload designs with on-demand, tunable pharmacokinetics and minimized off-target accumulation [1,5].

Advanced ligand engineering utilizes multivalency and the spatial organization of the ligand shell to regulate receptor engagement and downstream signaling. Tutorial and review articles on inorganic nanoparticle functionalization explain the construction of multi-layered or orthogonal ligand shells by sequential chemistries or click reactions to integrate stealth polymers, small-molecule ligands, and biological macro-ligands without disrupting colloidal stability [103,105]. In the case of imaging nanoprobes, multivalent presentation of targeting ligands (e.g., RGD peptides, folate, or antibodies) can offer a higher degree of avidity and give rise to receptor clustering that can, in turn, enhance cellular uptake and amplify local contrast effects, especially for low-abundance targets [3,5,107]. Conversely, overdense ligand packing can accelerate opsonization or impede the penetration of water to paramagnetic centers, resulting in lower relaxivity and requiring quantitative tuning of ligand packing density and direction [3,103]. Integration of responsive linkers into ligand architectures further enables the shedding of stealth layers or the exposure of otherwise masked targeting epitopes within disease-associated microenvironments, providing a bridge between static surface engineering and the dynamic behaviors discussed in Section 3.2 [96,108].

### 3.2. Stimuli-Responsive and Smart Carrier Designs

Stimuli-responsive nano-contrast carriers are designed to undergo physicochemical changes in response to endogenous factors (pH, redox potential, enzymes, hypoxia, reactive oxygen species) or external stimuli (light, heat, ultrasound, magnetic fields), thereby coupling the imaging signal and payload release to specific biological conditions. Comprehensive reviews classify such systems based on trigger type and highlight polymeric, lipidic, and inorganic architectures that incorporate cleavable linkers, charge-switching moieties, solubility transitions, or structural disassembly motifs [96,97,109]. In oncology, taking advantage of the acidic tumor environment, which is rich in enzymes, redox-active species, and glutathione, pH- and glutathione-responsive carriers have been used to unmask the contrast payloads or therapeutic agents selectively at tumor sites, minimizing the background signal and toxicity to the rest of the body [96,109,110]. For imaging, such responsiveness can be revealed as off-to-on fluorescence activation, turn-on T1 MRI contrast upon ion release, or aggregation-induced changes in photoacoustic or CT signals [13,96,108]. These behaviors turn nano-contrast agents from passive reporters into probes that dynamically report local biochemical information in their signal profiles, enhancing specificity and functional readout capacity.

A particularly active area is that of activable multimodal nanoprobes that combine multiple signal channels within a single stimuli-responsive platform. Yang et al. provide numerous examples of tumor microenvironment cues, such as acidic pH, overexpressed proteases, or elevated glutathione, that serve as stimuli for simultaneous activation of MRI, optical, CT, or photoacoustic signals, often in combination with controlled drug release [108]. In such designs, cleavage of a linker or dissolution of a responsive shell can simultaneously unquench fluorophores, release paramagnetic or high-Z ions, and free chemotherapeutics, and enable real-time monitoring of drug distribution and microenvironmental parameters [96,108]. For example, redox-sensitive silica or polymeric nanocarriers that release Mn^2+^ or Fe^3+^ under tumor-reducing conditions can switch from low- to high-T1 contrast and catalyze Fenton- or Fenton-like reactions for chemodynamic therapy [96,111]. Multi-compartment carriers go even further by enabling the separation of imaging and therapeutic modules across different domains, with stimulus-induced structural changes altering the kinetics of signal and release in a controllable, hierarchically programmed way [10,108].

Beyond single-trigger designs, smart carriers are going further, exhibiting logic-like behavior that requires sequential or combined stimuli to activate imaging or therapy. Reviews on stimuli-responsive nanocarriers focus on multistage systems that first reduce their size or surface charge to enable tumor penetration and then respond to intracellular stimuli to release their payloads, often in combination with real-time tracking signals [96,109]. Smart liposomes and polymeric micelles activated by hyperthermia, ultrasound, or light exhibit heat- or energy-induced structural changes that accelerate drug release while simultaneously improving local contrast for monitoring treatment [97,109]. These ideas have been expanded into nano-contrast carriers that emit a specific signal only when both a disease biomarker and an externally applied stimulus are present, thereby enhancing discrimination between diseased and healthy tissue. In parallel, microenvironment-informed designs that incorporate metabolic or redox sensors into the carrier matrix promise dynamic measurement of therapy-induced changes, enabling closed-loop imaging-guided interventions rather than single-time-point diagnostics [96,107,110].

### 3.3. Multimodal and Multi-Responsive Platform Design

Multimodal nano-contrast carriers integrate two or more imaging modalities into a single architecture to leverage complementary strengths in sensitivity, spatial resolution, and quantitative accuracy. Reviews of molecular imaging nanoprobes and (nano)probe-based diagnostics emphasize that combining, for example, MRI with PET, CT, optical, or photoacoustic imaging can yield both anatomic and molecular information to better characterize the lesion and aid in treatment planning [3,107,112]. In many systems, a high-relaxivity MRI core (e.g., iron oxide or manganese oxide) is colocalized with high-Z elements for CT, radionuclides for PET/SPECT, or fluorophores for intraoperative visualization within a single carrier to ensure spatially congruent signals [1,3,113]. Magneto-optical nanosystems are a good example of this trend, as they combine magnetic elements for MRI with plasmonic or upconversion elements for optical and photoacoustic imaging, enabling in-depth, real-time guidance of thermal or photodynamic therapies [13,114]. From a design perspective, the challenge is to balance payload ratios and the spatial arrangement of these components to avoid mutual signal quenching while maintaining good biocompatibility and pharmacokinetics [107,112,114].

A multifunctional material that serves as a dual contrast agent, rather than merely a passive carrier for separately loaded reporters, provides another layer of multifunctionality. Iron oxide nanoparticles provide a good example of this, as they can switch from T2-dominant to T1-dominant depending on particle size (from superparamagnetic to ultrasmall), and even achieve a tunable dual T1-T2 response in engineered formulations. The controlled size and composition of manganese-engineered iron oxides also demonstrate modulation of both relaxation channels on a single platform [115,116,117]. This concept is extended by lanthanide-based upconversion nanostructures that allow simultaneous NIR-to-visible upconversion emission and the inclusion of MRI-relevant dopants such as Gd or other paramagnetic lanthanides, which can support optical, MRI, and sometimes CT or nuclear imaging workflows in a single nanostructure [118,119]. Another canonical example is given by gold nanoparticles, which have strong CT attenuation and photoacoustic or optical contrast without the need for any additional payload loading [120,121].

At the design level, these properties are often introduced into these materials via three channels. Firstly, the incorporation of elemental dopants or other atoms introduces paramagnetic or radiopaque centers into a stable crystal lattice, as in manganese-based and lanthanide-based systems. Second, through structural engineering, particularly control of particle size, clustering, and core–shell architecture, the balance of T1 and T2 relaxation or the preservation of separate signal channels in a single construct can be altered. Third, surface functionalization can provide an additional imaging dimension by attaching a second imaging component, such as chelated radionuclides, fluorophores, or targeting ligands, as a means to combine CT/MRI, CT/optical, or PET/MRI on an intrinsically contrast-enhancing core [115,116,117]. The inherent X-ray attenuation properties of high-Z materials make them useful as CT agents, while their surfaces can be further modified for multimodal applications and quantitative spectral imaging [122,123]. In this context, next-generation multifunctional nano-contrast systems are not just carriers of contrast agents, but also contrast-active materials whose chemistry and architecture are carefully designed to achieve the desired imaging function [1,119,124].

Structurally, multimodal platforms often use core–shell, yolk–shell, or Janus designs that keep distinct functional domains separate while remaining mostly nanoscale in size. Silica-based carriers remain a versatile chassis, as they can host paramagnetic ions, dyes, and radiosensitizers in distinct compartments of the porous framework while allowing independent tuning of pore size, shell thickness, and surface chemistry [10]. Examples include Gd-chelated silica nanospheres integrating chemo- and photothermal agents for combined MRI, thermal ablation, and chemotherapy, or hollow mesoporous silica shells loaded with perfluorocarbon and decorated with magnetic cores for integrated MR/ultrasound imaging and image-guided drug delivery [10,12,114]. Parallel developments in nanosystems based on manganese oxides and iron oxides are showing how suitable control of crystal structure, doping, and surface coating can result in single particles that can be used for both T1/T2 MRI and other imaging modalities, such as photoacoustic or CT imaging, while maintaining acceptable relaxivities and toxicity profiles [111,113]. Nuclear medicine-oriented theranostic platforms further overlay PET or SPECT functionality using chelated or native radionuclides, thereby enabling quantitative whole-body tracking of these complex transporters [3,12].

Multi-responsive multimodal platforms combine stimuli-sensitive design elements within carriers that already include multiple imaging readouts. Activatable nanoprobes, as described in the tumor theranostics literature, demonstrate the advantage of pH-, enzyme-, or redox-induced changes that simultaneously improve MRI relaxivity, reverse fluorescence quenching, and modify the photoacoustic signal while triggering drug release [13,108,111]. In magneto-optical nanosystems, external fields offer an extra dimension of control, for example, magnetic targeting or hyperthermia, with corresponding changes in MRI and optical contrast reporting on local heating and therapeutic progress [113,114]. Reviews of smart MRI-based theranostics highlight that such systems can be designed to provide imaging signals that directly reflect the therapeutic mechanism, for example, via chemodynamic therapy-induced changes in T_1_/T_2_ contrast or reactive oxygen species (ROS) that activate the probe [113]. When combined with surface architectures that allow modular exchange of targeting ligands or payloads, these multi-responsive design approaches provide a flexible pathway to disease- and patient-specific customization of probes, at the expense of added synthetic complexity and greater characterization requirements [107,108,109].

### 3.4. Targeting Strategies and Personalization by Design

Targeting strategies for nanocarriers traditionally distinguish between passive accumulation, driven by the enhanced permeability and retention effect, and active targeting via ligand-mediated recognition of overexpressed receptors or other biomarkers. For these reasons, contemporary reviews focus on various physicochemical parameters (size, shape, surface charge) and on the selection and design of ligands (e.g., peptides, antibodies, aptamers, or small molecules) to maximize tumor targeting relative to healthy tissues [110,125,126,127]. Reviews of ligand-based active targeting strategies list numerous receptor–ligand pairs, including folate, integrins, transferrin, and growth factor receptors, that have been successfully exploited in theranostic nanomedicines, while also noting challenges such as receptor heterogeneity and competition with endogenous ligands [126,127]. Broader analyses of targeted drug delivery reveal parallel strategies for delivery via physical and cell-mediated methods, such as magnetic guidance, focused ultrasound, and carrier systems that use immune or stem cells as delivery shuttles [110,113,125]. For nano-contrast carriers, such approaches will have to be harmonized with imaging readouts to make signal intensity indicative not only of carrier presence but of successful engagement of intended molecular targets.

Personalization by design builds on population-level targeting based on biomarkers and extends to patients’ molecular signatures and disease phenotypes. Recent molecular imaging and nanomedicine reviews argue that the development of nanoprobes should be guided more by quantitative information on receptor expression, tumor microenvironment features, and immunological status, gathered from genomics, pathology, and imaging, to enable rational selection or combination of ligands for each indication [3,107,110]. In this context, the design and development of modular carrier systems with interchangeable ligand and payload slots are of particular interest, as they can be reconfigured to target different targets while preserving a validated core chemistry and pharmacokinetic profile [1,12,125]. Multimodal imaging nanoprobes also support the personalization approach by delivering coregistered anatomical, functional, and molecular data that can be fed into AI-assisted analysis pipelines to stratify patients, predict response, and iteratively refine probe design [107,113]. Nuclear theranostic platforms, in which the same or closely related carriers are used for diagnostic imaging and targeted radionuclide therapy, are a good example of how contrast agents can be incorporated into therapeutic decision-making workflows by aligning nano-contrast carrier engineering with precision oncology paradigms [4,12].

## 4. Theranostic and Biological Applications

**Figure 5:**
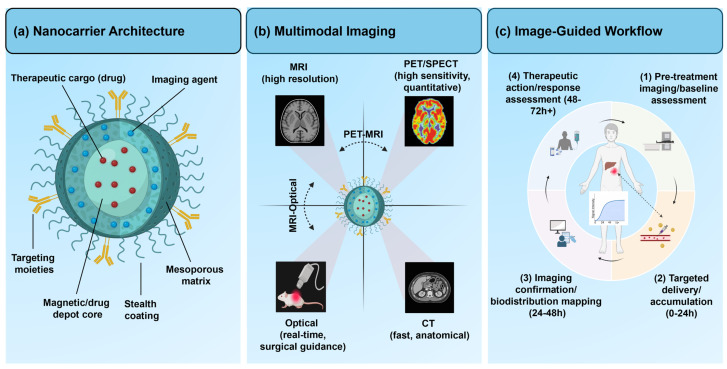
Conceptual design and clinical use logic of multifunctional nano-contrast carriers. (**a**) Representative nanocarrier architecture. (**b**) A single carrier can be engineered for multimodal imaging by incorporating reporters compatible with MRI (high spatial resolution), PET/SPECT (high sensitivity and quantitation), CT (anatomical context), and optical imaging (real-time intraoperative guidance), which enables cross-validated localization and quantification. (**c**) Example image-guided workflow in which baseline imaging precedes administration, followed by early accumulation assessment (0–24 h), biodistribution confirmation (24–48 h), and longitudinal evaluation of therapeutic response (≥48–72 h) to support adaptive, feedback-informed interventions. Partially created in BioRender. Huh, Y. S. (2026). https://BioRender.com/qughrx8, accessed on 20 June 2026.

Theranostic nano-contrast carriers combine imaging, targeting, and therapeutic functions within a single nanoscale construct to enable the characterization of disease, the delivery of treatment, and the monitoring of response along a common pharmacokinetic trajectory [105,106]. Compared to conventional contrast agents, nanostructured systems can provide improved payload capacity, tailor circulation, and enable the combination of different imaging modalities with different therapeutic mechanisms, including chemotherapy, radiotherapy, and immune modulation [2,4]. At the same time, multifunctional carriers underscore the importance of biological context, as the same surface motifs and microenvironment-responsive features that mediate imaging signals often control cellular uptake, intracellular trafficking, and therapeutic action [1,128]. Within the framework developed in the previous sections on carrier design and targeting, the present part of the review discusses the ways in which the development of dendrimer, liposome, polysaccharide, and silica-based platforms has been directed toward programmable theranostic systems and how their interactions with tissues and immune cells can be exploited for biologically informed molecular imaging. Figure 5 summarizes the theranostic applications discussed in this section.

### 4.1. Theranostic Applications

Theranostic nanocarriers are generally structured based on a core scaffold that coassembles contrast reporters, therapeutic payloads, and targeting or stimuli-responsive elements, often in separate but communicating domains [129,130]. Recent reviews focus on design strategies that vary between polymeric and liposomal constructs with covalently linked drugs and chelated metal ions, and inorganic and hybrid platforms that confine radiosensitizers, photosensitizers, or gene vectors with multimodal imaging labels [128,131]. A common theme is the use of the carrier to synchronize drug exposure and imaging readouts in space and time, enabling quantitative assessment of intratumoral distribution, target engagement, and early biological response. Activatable architectures, in which the release of therapy and the enhancement of contrast are both induced by a single pH, enzyme, or redox cue, further reduce the time between diagnosis and intervention, enabling feedback-controlled treatment schemes [108,132]. A large class of theranostic applications includes image-guided chemotherapy and radiotherapy, in which nanocarriers concentrate the drug payload (cytotoxic or radionuclide) within diseased tissues while simultaneously reporting on biodistribution. Polymeric micelles, dendrimers, and liposomes have been used to encapsulate small-molecule chemotherapeutics, along with MRI, CT, or optical reporters, to improve tumor accumulation and enable longitudinal monitoring of drug deposition and washout [133,134]. Iron oxide and other magnetic nanoparticles form the basis of MRI-guided treatments, in which these nanoparticles serve as contrast agents, drug depots, and, in some cases, local hyperthermia mediators [135,136]. Radiolabeled nanogels and other soft nanomaterials extend this idea to nuclear medicine, enabling PET or SPECT imaging platforms that can be converted into targeted radiotherapeutics by switching isotopes or doses, embedding dosimetry, and integrating efficacy assessment into the carrier design itself [137,138]. Energy-based nanotheranostics use optical, acoustic, or magnetic energy to simultaneously couple local signal amplification with physical or photochemical tumor ablation. Gold, carbon, and upconversion nanostructures that act as photothermal or photodynamic transducers, as well as optical, photoacoustic, or CT contrast agents, are good examples of this approach and support real-time visualization of light distribution, temperature rise, and treatment margins [139,140,141]. Magneto-optical nanosystems consist of a magnetic core and a plasmonic or luminescent shell, enabling MRI, optical, and photoacoustic tracking of field-guided or magnetothermal therapies within a single carrier architecture [114,142]. More recently, biosynthesized magnetosomes tagged with near-infrared dyes and immunomodulators have been developed to achieve both MRI and fluorescence guidance, and to induce immunogenic cell death and macrophage repolarization, blurring the boundary among local ablation, systemic immune priming, and molecular imaging readout [143].

### 4.2. Multimodal and Multi-Parametric Imaging

Multimodal imaging is an approach intended to overcome the limitations of single-modality readouts by integrating complementary modalities, and nano-contrast carriers are natural vehicles for such integration because they can colocalize heterogeneous reporters within a single pharmacokinetic entity [107,144]. Nanoparticles that include paramagnetic metals for MRI, high atomic number elements for CT, radionuclides for PET or SPECT, and fluorophores for optical or photoacoustic imaging have been developed in various configurations to enable simultaneous acquisition of high-resolution anatomical, functional, and molecular information [145,146]. Radiolabeled inorganic and polymeric nanostructures are of particular interest because they enable hybrid PET/SPECT-CT and PET/MRI systems for quantitative, whole-body tracking of carrier distribution and target engagement, and for cross-validation of signals with structural imaging [147,148].

Beyond modality combinations, theranostic carriers are increasingly incorporated into multiparametric imaging, in which a single modality, such as MRI or PET, provides multiple quantitative maps of tissue structure and physiology. Iron oxide and other magnetic nanoparticles can be engineered to affect T_1_-, T_2_-, and susceptibility-weighted MRI, which, in parallel, probe vascular permeability, cellularity, and microvascular architecture [149]. Advanced MRI protocols incorporating diffusion, perfusion, and metabolic sequences permit nanoparticle-enhanced acquisitions to be converted into parameter sets that reflect perfusion, extracellular volume, apparent diffusion, and, in some instances, pH or oxygenation, providing richer input for radiomics and assessment of response to therapy [150,151]. In oncology, radiolabeled theranostic carriers further extend multiparametric analysis by linking PET-derived kinetic parameters and receptor occupancy with coregistered CT or MRI features, facilitating voxel-wise correlation of drug exposure, signaling pathway modulation, and structural response [152,153]. Table 3 summarizes common multimodal imaging combinations in nano-contrast systems and illustrates how distinct integration strategies enable coregistered anatomical and molecular readouts.

### 4.3. Immune System Interactions and Monitoring

**Figure 6:**
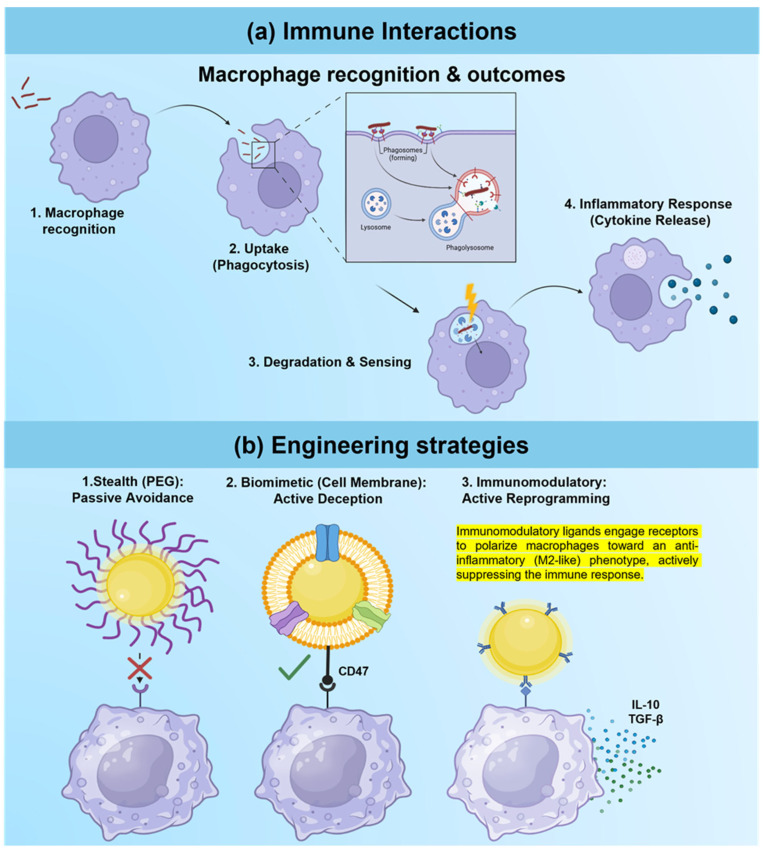
Innate immune processing of nano-contrast agents and surface engineering strategies to modulate it. (**a**) Schematic of macrophage-mediated recognition, phagocytic uptake, intracellular degradation/sensing, and downstream inflammatory signaling (cytokine release) that collectively influence clearance and toxicity. (**b**) Representative engineering strategies to tune immune interactions: (i) PEG stealth to reduce opsonization and uptake, (ii) biomimetic cell membrane camouflage (e.g., signals such as CD47) to reduce macrophage recognition, and (iii) immunomodulatory ligands that actively reprogram macrophage phenotype and cytokine outputs to support therapeutic goals while preserving imaging performance. Partially created in BioRender. Huh, Y. S. (2026). https://BioRender.com/e5oencx, accessed on 20 June 2026.

Because nano-contrast carriers inevitably interact with innate and adaptive immune components, their use in biological applications is closely linked to immunological mechanisms. Systematic analyses demonstrate that nanoparticle size, surface charge, shape, and composition determine complement activation, opsonization, and uptake by monocytes, macrophages, and dendritic cells, which in turn affect biodistribution, clearance, and cytokine release profiles [163,164]. Protein corona formation on nano-contrast agents further modulates recognition by immune receptors; corona composition, particularly when enriched in immunoglobulins and complement factors, can either exacerbate inflammation or promote tolerance depending on its makeup and dynamics [165]. These insights have led to attempts to create stealthy or immune-interactive surfaces on imaging carriers, for example, via coatings with zwitterionic, biomimetic, or checkpoint ligands, with the dual purpose of improving imaging performance and reducing adverse immune responses [166,167]. As shown in Figure 6a, macrophage recognition and phagocytic clearance can simultaneously limit the imaging window and drive inflammatory risk. Accordingly, surface designs spanning PEGylation, biomimetic membrane camouflage, and immunomodulatory ligand display are increasingly used to reduce opsonization or actively tune immune engagement (Figure 6b).

At the same time, nano-contrast systems are increasingly used as tools in immune monitoring, which has led to the development of cancer immunoimaging. Smart nanoparticles that selectively bind to or are internalized by T cells, dendritic cells, tumor-associated macrophages, or checkpoint receptor-expressing cells have been labeled with optical, MRI, and nuclear reporters to visualize immune cell trafficking, activation, and spatial organization within tumors and lymphoid organs [70]. Recent reviews cover imaging strategies for following cell-based immunotherapies, such as engineered T cells and natural killer cells, using nanoparticle labels that allow repeated in vivo observation without affecting cell viability or function [168,169]. Molecular imaging probes targeting immune checkpoint expression, cytokine environment, or effector cell infiltration are also being coupled to theranostic carriers, enabling simultaneous assessment of tumor burden, immune landscape, and therapeutic modulation during a single imaging session [107,170].

A further evolution is the development of immunomodulatory theranostic carriers that can actively shape immune responses while also reporting on their own effects. Nanomaterials have been used to manipulate macrophage metabolism and polarization, such as to shift the metabolic state of tumor-associated macrophages from an immunosuppressive M2 phenotype toward a proinflammatory M1 phenotype, with imaging labels embedded to monitor carrier accumulation and microenvironmental changes over time [171,172]. As illustrated by magnetosome-based systems that combine MRI/fluorescence with photo-immunotherapy (see Section 4.1), such platforms can simultaneously report on immune cell recruitment while delivering immunogenic cell death [143,173]. Looking ahead, rationally designed nano-contrast carriers that couple finely tuned engagement of the immune system with quantitative readouts by imaging are expected to play an important role in personalization of immunotherapies, provided that long-term immunotoxicity and off-target immune perturbations are rigorously characterized [164,169].

## 5. Biodistribution, Pharmacokinetics, and Safety Considerations

The clinical value of multifunctional nano-contrast carriers depends not only on imaging sensitivity and multifunctionality but also, critically, on their in vivo distribution, persistence, transformation, and elimination. Unlike small-molecule contrast agents, nanoformulations exhibit complex, nonlinear pharmacokinetics that are influenced by carrier size, morphology, surface chemistry, and payload, resulting in highly organ- and microenvironment-specific accumulation patterns. Comprehensive characterization of absorption, distribution, metabolism, and excretion (ADME) is therefore important for balancing imaging performance with long-term safety and for rationally comparing the performance of traditional dendrimer-, liposomal-, chitosan-, and silica-based platforms with emerging theranostic systems. General reviews of nanomedicine pharmacokinetics and biodistribution point out that nanobio interactions at the blood, endothelial, and tissue barriers can radically alter exposure profiles relative to the parent drugs, such that nano-specific evaluation frameworks must replace simple extrapolation from conventional agents [174,175]. Simultaneously, accumulating evidence of organ-specific toxicity induced by inorganic nanomaterials used for imaging has highlighted the need to integrate biodistribution, pharmacokinetics, and toxicology into a single safety-by-design paradigm for nano-contrast carriers [176,177].

### 5.1. Classical Determinants of Biodistribution

**Figure 7:**
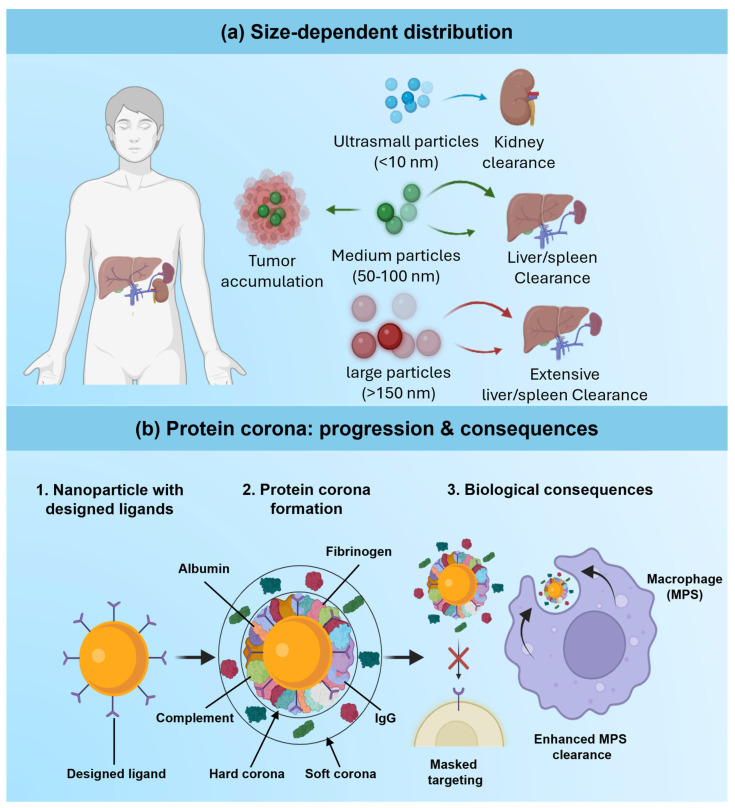
Key biological determinants of nano-contrast carrier fate: size and corona formation. (**a**) Size-dependent biodistribution: ultrasmall particles (<10 nm) preferentially undergo renal filtration; intermediate sizes (~50–100 nm) favor prolonged circulation and tumor accumulation; and larger particles (>150 nm) show increased hepatic/splenic sequestration and clearance. (**b**) Protein corona formation on engineered nanoparticles and its consequences: adsorption of plasma proteins creates a new biological identity that can mask designed ligands, alter targeting, and promote opsonization and macrophage uptake, thereby reshaping circulation time and organ accumulation. Partially created in BioRender. Huh, Y. S. (2026). https://BioRender.com/bh3ftjh, accessed on 20 June 2026.

Classically, hydrodynamic size, shape, and surface charge are the key factors that determine nanoparticle biodistribution, circulation half-life, vascular extravasation, and clearance pathways. Renal filtration has a stringent size cutoff (about 5–6 nm for rigid spherical particles); therefore, ultrasmall carriers like quantum dots or gold clusters that are smaller than this cutoff are quickly eliminated from the body, while larger carriers are eliminated mainly by hepatobiliary and mononuclear phagocyte system (MPS) pathways (Figure 7a) [86,178]. Tumor concentration of nano-contrast agents is generally described by the EPR effect, which favors long-circulating particles in the 20–200 nm size range; however, clinical data indicate substantial inter- and intrapatient heterogeneity in the magnitude and spatial patterning of the EPR effect [179,180]. For dendrimer-, liposome-, chitosan-, and silica-based contrast agents, tuning core size, rigidity, and aspect ratio therefore remains a first-order design handle to shift distribution between blood pool, tumor, liver, spleen, and kidney, but has to be balanced against constraints on payload loading and imaging sensitivity [9,10,28,66].

Beyond size and charge alone, surface chemistry and the adsorbed protein corona are major regulators of the fate of nanocarriers and constitute the true biological identity of the particle that is recognized by complement, opsonins, and cell receptors (Figure 7b). Recent work has systematically dissected the influence of nanoparticle size, curvature, hydrophobicity, and ligand patterning on the composition and dynamics of corona structure, with downstream consequences for circulation time, organ uptake, and immune recognition [181,182]. Stealth coatings like PEG or polymers with both positive and negative charges (zwitterionic polymers), while reducing nonspecific protein adsorption and MPS uptake, may also mask targeting ligands and, in some patients, elicit anti-PEG immune responses, changing pharmacokinetics across repeat dosing [151,154]. In practice, for nanoparticles to be useful, the surface must be engineered not only for colloidal stability and ligand presentation but also for the predictable formation of a corona in clinically relevant biofluids.

Classical biodistribution patterns also reflect organ-level anatomical and physiologic filters. The liver and spleen are major sites of sequestration by resident macrophages and fenestrated sinusoids for many nano-contrast agents, especially for particles above about 100 nm or with opsonizing coronas, while renal clearance is exquisitely sensitive to size, charge, and deformability at the glomerular barrier [174,178,183,184]. Mesoporous silica nanoparticles, for example, exhibit biodistribution that is strongly influenced by pore structure, degree of condensation, and PEGylation, with many formulations showing predominant hepatic uptake and variable renal excretion, depending on size and degradation rate. Iron oxide and gold nano-contrast agents also have a propensity to accumulate in the liver and spleen, and biodistribution is influenced by core size, magnetic properties, and surface coatings [177,185].

The chemical identity of the carrier core is a primary determinant of long-term fate, in addition to the size and surface-chemistry effects mentioned above. Iron oxide cores undergo the normal iron-handling processes of the body, where they, after being picked up by macrophages of the mononuclear phagocyte system in liver and spleen, are broken down and the liberated iron fed into transferrin-mediated transport for reuse in hemoglobin synthesis, a process which generally results in favorable long-term tolerability [56,186]. The magnetic properties of intact iron oxide cores, however, persist until complete degradation, causing local field inhomogeneities that produce T2/T2* signal voids; this feature has been exploited for cell tracking but complicates the interpretation of long-term organ retention [56,187]. By contrast, gold cores are bioinert and do not break down; they simply accumulate progressively in Kupffer cells and splenic macrophages, where they can persist for months without evidence of breakdown, raising questions about chronic organ burden even when acute hepatic toxicity markers remain normal [188,189].

Gadolinium-based cores and coatings have a unique risk profile for transmetallation, or the displacement of Gd(III) from its chelating environment by endogenous cations (e.g., zinc, copper, iron) to release free Gd(III) ions capable of depositing in bone, skin, and brain tissue, and, in patients with compromised renal clearance, causing nephrogenic systemic fibrosis [190]. This risk is greatly influenced by the stability of the chelating agent and the surrounding matrix, and the presence of free gadolinium ions is minimized by incorporating the ion into a more rigid matrix, such as polysiloxane or an inorganic nanoparticle framework. Silica-based cores also have another mode of hydrolysis where the siloxane framework is dissolved into soluble, renal-excreted silicic acid; the rate of this hydrolysis is highly dependent on the porosity, surface area, and degree of condensation of the siloxane framework, with loose, high-surface-area mesoporous structures degrading significantly faster than dense, highly condensed solid silica cores [62,191]. These contrasting core-dependent mechanisms suggest that core composition should be considered a key design parameter for controlling long-term biodistribution and safety, rather than merely a secondary effect of size and coating.

### 5.2. Advanced Pharmacokinetic Modeling

Traditional noncompartmental pharmacokinetic analysis is often insufficient for nanocarriers because it cannot explicitly account for carrier–tissue interactions, nonlinear uptake in the MPS, or depot effects in tumors and lymphatics. Recent reviews emphasize that nanomedicine ADME emerges from a hierarchy of scales, in which mechanistic models are indispensable for understanding and predicting nanocarrier behavior [175,180]. Physiologically based pharmacokinetic (PBPK) frameworks have therefore been modified to include permeability-limited compartments, explicit MPS uptake, and multi-route administration, thereby enabling simultaneous fitting of blood and multi-organ time–concentration data for various nanoplatforms [192,193]. For multifunctional contrast agents, these models can be further extended to separately trace the carrier, payload, and imaging signal, thereby clarifying the effects of design choices on both diagnostic and therapeutic exposure.

Recent progress in PBPK modeling of nanoparticles, in particular, has focused on standardizing model structures, clarifying when perfusion- or permeability-limited representations are suitable, and integrating high-content experimental data. Kutumova et al. [192] provide a detailed overview of existing nanoparticle PBPK models and associated software tools, discussing how model complexity can be adjusted to the scientific question, ranging from very basic models with 5 compartments to whole-body, tumor-bearing models. Tutorial-like work has further simplified the design of PBPK models for nanoparticles, and best practices for route-specific coronas, endocytic uptake, and interspecies scaling have been codified [194]. Clinical translation case studies demonstrate the use of nanoparticle PBPK models to rationalize the in vivo fate of targeted formulations, such as nanocrystal anticancer agents or long-acting depot systems, as well as for dose selection and risk assessment [193,195].

### 5.3. Enhanced Safety Profiling

Safety concerns for nano-contrast agents have been most apparent with gadolinium-based contrast agents (GBCAs), where nephrogenic systemic fibrosis in renally impaired patients and evidence of long-term gadolinium retention in the brain and bone have led to intense scrutiny. Mechanistic reviews emphasize several mechanisms of gadolinium toxicity, including transmetallation, oxidative stress, and the precipitation of insoluble gadolinium-containing deposits in tissues, with risk strongly dependent on chelate structure (linear vs. macrocyclic) and cumulative dose [196]. These concerns have stimulated the development of nano-based approaches for immobilizing gadolinium into more stable matrices or replacing GBCAs with iron oxide, manganese, or other inorganic nano-contrast platforms that have similar relaxivity with less free-metal exposure [176,185].

For inorganic nano-contrast agents themselves, abundant toxicology data show complex, dose- and design-dependent behavior rather than uniform hazard. Iron oxide nanoparticles, widely investigated for MRI, cell tracking, and theranostics, have generally been shown to be safe at clinically relevant doses, but some formulations and coatings can cause oxidative stress, genotoxicity, or developmental effects in preclinical models [197]. Similarly, studies of silica-based nanoparticles reveal that biodegradation rate, porosity, and surface functionalization are of vital importance for inflammation, organ retention, and excretion; substantial hepatic clearance and gradual dissolution to silicic acid are observed for many mesoporous silica systems, yet both poorly degradable and highly cationic forms can result in long-term tissue responses [183,198]. Gold nanoparticles as CT or optical/PET contrast enhancers are an example of how platform-specific design (core size, shape, ligand type, and dose) controls bioaccumulation and toxicity, and therefore needs careful formulation-by-formulation assessment [177].

Enhanced safety profiling of nano-contrast carriers is gradually shifting away from simple cytotoxicity assays and acute organ histology toward integrated, tiered strategies that combine in vitro, in vivo, and in silico evidence. Contemporary nanotoxicology reviews focus on mechanistic endpoints such as oxidative stress, DNA damage, inflammasome activation, and interference with cytochrome P450 systems, in addition to traditional clinical chemistry and histopathology, to anticipate long-term risks [176,199,200]. Regulatory-oriented overviews of nanomedicine safety emphasize the importance of case-by-case evaluation, standardized characterization, and careful extrapolation from animal to human studies, especially for complex theranostic constructs with multiple active components [199]. As multifunctional nano-contrast platforms advance toward precision theranostics, integrating PBPK modeling, longitudinal imaging of biodistribution, and advanced immunotoxicity testing into development pipelines will be key to de-risking clinical translation and to informing robust benefit–risk assessments for next-generation carriers [192,196].

## 6. Clinical Translation

The clinical development of multifunctional nano-contrast carriers has lagged behind the rapid growth of preclinical nanotheranostic research. Although there are now dozens of approved nanomedicines, only a handful of them actually serve as contrast agents or as theranostics, that is, molecular imaging platforms, while an even smaller number of them have true multimodal or stimuli-responsive functionalities [201,202]. Systematic analyses highlight that translation is often blocked not by a lack of efficacy in animal models, but by manufacturing complexity, insufficient characterization, uncertain regulatory classification, and limited economic viability [21,203,204]. Recent frameworks, like the DELIVER guideline, focus on the clinical success that will require integration of the target product profile, scalable process design, robust pharmacology, and business strategy from the earliest stages of formulation design [21]. Market-focused overviews likewise demonstrate that nanoformulations that reuse clinically familiar materials (e.g., lipids, iron oxides, gadolinium chelates) and address a well-defined clinical problem are overrepresented among approved products [202,204]. These lessons are the context in which multifunctional nano-contrast carriers need to be evaluated and optimized for translation.

### 6.1. Current Clinical Status

**Figure 8:**
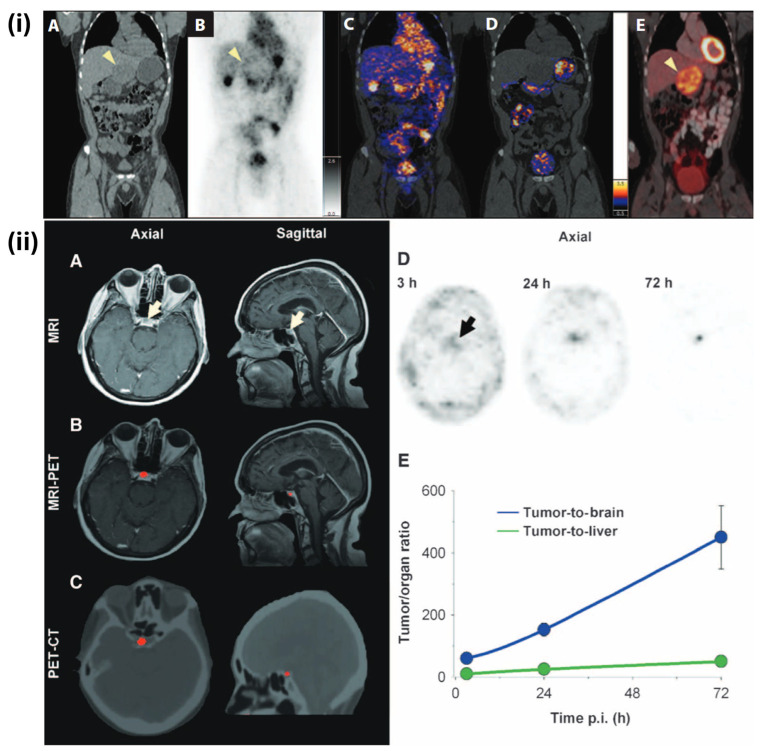
Clinical PET-based tracking and multimodal imaging of ^124^I-cRGDY–PEG–C dots. (**i**) Whole-body PET/CT in a patient with a hepatic metastasis: (**A**) Reformatted coronal CT demonstrates a hypodense left hepatic lobe metastasis (arrowhead); (**B**) Coronal PET image at 4 h post-injection demonstrates particle activity along the peripheral aspect of the tumor (arrowhead), in addition to the bladder, gastrointestinal tract, gallbladder, and heart; (**C**) Co-registered PET/CT at 4 h post-injection localizes activity to the tumor margin; (**D**) Co-registered PET/CT at 24 h post-injection localizes activity to the tumor margin; (**E**) Corresponding 18F-FDG PET/CT image showing the hepatic metastasis (arrowhead). Color and gray scales reflect SUV values. (**ii**) Multimodal imaging of a pituitary lesion: (**A**) Multiplanar contrast-enhanced MR axial and sagittal images at 72 h post-injection demonstrate a subcentimeter cystic focus (arrows) within the right aspect of the anterior pituitary gland; (**B**) Co-registered axial and sagittal MRI–PET images reveal increased focal activity localized to the lesion site; (**C**) Axial and sagittal PET/CT images localize activity to the right aspect of the sella; (**D**) Axial PET images of the brain at 3, 24, and 72 h post-injection demonstrate progressive accumulation of activity within the sellar region; (**E**) Tumor-to-brain and tumor-to-liver activity ratios as a function of post-injection (p.i.) time. Adapted with permission from ref. [16]. Copyright 2014, American Association for the Advancement of Science.

Clinically advanced nano-contrast and theranostic carriers are still concentrated in a few families of materials, with a focus on liposomal drugs, iron oxide formulations, and high-Z inorganic platforms, of which only a few examples are designed as imaging-guided theranostic agents [201,202]. Ultrasmall silica Cornell dots have been among the first examples of an inorganic–polymer hybrid probe that has successfully passed the first-in-human trial of targeted PET–optical imaging of metastatic melanoma with renal clearance and positive dosimetry for ^124^I-cRGDY-PEG-C dots (Figure 8) [16]. Gadolinium-based AGuIX^®^ nanoparticles, which consist of a polysiloxane matrix, have advanced from preclinical studies with radiosensitizing properties to early clinical evaluation; the AGuIX^®^ nanoparticles are used intravenously as MRI-visible radiosensitizers in combination with radiotherapy [17]. Phase I work in patients with multiple brain metastases (NANORAD) established acceptable safety and pharmacokinetics and suggested enhanced local control when AGuIX was combined with whole-brain irradiation [17,205]. More recently, adaptive trials such as NANO-GBM have expanded the use of AGuIX to newly diagnosed glioblastoma by using the nanoparticles in combination with concurrent chemoradiation [20].

Beyond these flagship platforms, the use of iron-based nanocarriers is a good example of how clinically familiar components can drive the translation of nonstandard contrast agents. A U.S. Food and Drug Administration (FDA)-approved intravenous iron supplement, ferumoxytol, has been widely used off-label as a blood-pool and cell-tracking MRI contrast agent and, more recently (October 2025), received FDA approval for a brain MRI indication [18,206]. Dedicated reviews of ferumoxytol-enhanced MRI (FE-MRI) report unique kinetic qualities, long intravascular residence, and high relaxivity for use in applications ranging from angiography to inflammatory and oncologic imaging [19]. Parallel work on the design of nanoparticle-based MRI contrast agents highlights the importance of size, surface charge, core composition, and ligand architecture in controlling pharmacokinetics, target accumulation, and image contrast, as well as in determining manufacturability and regulatory risk [207]. Table 4 summarizes representative nanocarrier and theranostic platforms that have advanced to human studies or regulatory approval. Together, these clinically used or trialed systems provide important proof of concept that multifunctional nanocarriers can meet human safety, imaging performance, and operational workflow requirements, even though approvals remain rare compared to preclinical studies [17,201,202].

### 6.2. Regulatory Pathway Evolution

Regulatory processes for nanocarrier theranostics are undergoing change amid the development and authorization of nanomedicine as a whole, but they remain fragmented, regionally and product-class-specific. Reviews of experiences worldwide indicate that nano-based products are typically assessed under current medicinal product or biologics regulations, with additional case-by-case scrutiny of nano-specific critical quality attributes, biodistribution, and immunotoxicity [204,208]. Multifunctional nano-contrast carriers often constitute combination products (drug-device or drug-diagnostic), leading to the involvement of several regulatory divisions and making it difficult to organize studies and dossier structure [21,203]. Comprehensive surveys show an increasing number of expectations by authorities regarding the justification of the intended clinical use (diagnostic versus theranostic), predefined imaging endpoints, and a mechanistic rationale for the links between nanoscale properties and clinical benefit and risk [203,208]. At the same time, guidance documents and scientific opinions now emphasize the need for platform comparability: minor differences in surface chemistry, ligand density, or payload can be considered a new product unless there are strong bridging data [204,208,209]. This platform- and product-specific stance is particularly relevant to modular nano-contrast carriers envisioned for repeated retargeting to different biomarkers.

**Figure 9:**
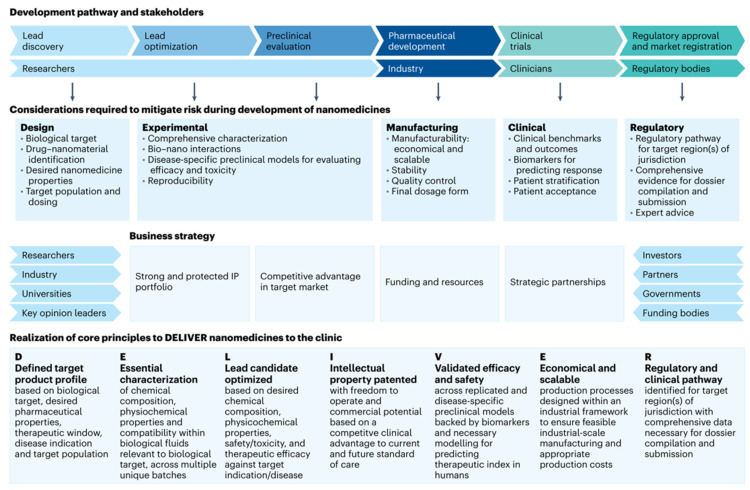
DELIVER framework and development pathway for clinical translation of nanomedicines. Adapted with permission from ref. [21]. Copyright 2024, Springer Nature.

In response, the regulatory science literature has shifted from listing obstacles to proposing structured roadmaps for nanomedicine and nano-enabled imaging agents. Hurdle analyses reveal the necessity of early determination of critical quality attributes, standardized multimodal characterization (physicochemical, in vitro, and in vivo), and statistically powered and clinically relevant models that better predict human pharmacokinetics and toxicity [204,208]. The DELIVER framework (Figure 9) explicitly recognizes the need to incorporate regulatory and business considerations into preclinical planning and argues that preclinical development should proceed only when a plausible regulatory path and package of evidence can be articulated from the outset [21]. Parallel reviews of specific nano-enabled modalities, such as nanoemulsions and inorganic high-Z platforms, report a growing demand from agencies for detailed impurity profiles, blood component interaction data, and long-term retention data, even when core materials are individually familiar [209]. Safe-by-design approaches that involve iterative refining of nanocarrier composition to minimize hazard while preserving function are recommended as a workable means of meeting emerging regulatory expectations [208,210].

### 6.3. Market Translation and Adoption

Even if regulatory approval were theoretically possible, the commercialization of multifunctional nanocarriers faces significant structural barriers. Market analyses have demonstrated that the vast majority of nanomedicines approved to date target large therapeutic markets (e.g., oncology, infectious diseases) with clear value propositions, whereas imaging-only agents face smaller reimbursable markets and intense competition from inexpensive small-molecule contrast media [202,211]. Reviews of clinical translation suggest that nano-theranostic platforms will need to demonstrate justification not only for incremental benefits in image quality, but also for measurable benefits in treatment selection, outcomes, or resource utilization to be adopted [203,212]. High manufacturing costs, batch-to-batch variability, and the need for specialized quality-control infrastructure further limit investor appetite for complex, multifunctional products [203,212]. Business-oriented analyses argue that many academically driven nanoplatforms lack realistic scalability and pricing strategies, leading promising agents to stall after early-phase trials due to an inability to justify the cost of pivotal studies relative to the expected market size [211,212].

Health economics and safety issues specific to contrast agents add another layer of complexity for market access. The experience with gadolinium-based contrast agents, in which concerns about tissue deposition and nephrogenic systemic fibrosis have led to labeling changes and the withdrawal of agents from the market, has sensitized payers and clinicians to long-term safety uncertainty [196,213]. Reviews note that even widely used small-molecule agents may face reimbursement issues due to a lack of clear evidence of added diagnostic value in high-quality trials [213]. For nanoparticle contrast carriers, long-term retention in the reticuloendothelial system or bone and possible immunological effects may lead to payer demands for postmarketing surveillance or risk management plans, with associated increases in the total lifecycle cost [196,210]. Consequently, market adoption often favors platforms that use biodegradable materials or fast-clearing components, or that reuse agents with robust human safety data (such as ferumoxytol), making both the regulatory and reimbursement stories easier to support [18,19,206,211].

Strategic frameworks for commercialization by design, therefore, advocate combining thinking about market access into scientific development from the beginning. Analyses of portfolios of nanomedicine products reveal that products that have a successful clinical course are often focused on a well-defined indication, are based on clinically well-known materials, and have clear differentiation with respect to the standard of care, such as the possibility to perform image-guided radiotherapy dose escalation or minimally invasive procedures that are not otherwise feasible [17,201,211]. Methodological papers highlight the need for early engagement with payers and health technology assessment bodies to ensure that trial endpoints align with reimbursement criteria, not only in terms of diagnostic accuracy but also in terms of impact on downstream management and costs [212]. From an industrial perspective, the development of modular nano-contrast platforms that can be produced via unified processes and then flexibly functionalized for different targets could spread fixed manufacturing costs across multiple indications, improving the business case [21,211]. Ultimately, the successful translation of multifunctional nano-contrast carriers will likely rely as much on coherent regulatory and market strategies as on advances in materials design and imaging physics.

## 7. Emerging Trends and Future Innovations

The next 10 years of nano-contrast agent research are likely to be characterized less by incremental advances on existing platforms and more by the convergence of advanced carrier chemistries, adaptive digital technology, and precision medicine workflows. Recent overviews of nanotheranostics focus on a transition from the development of single-material, single-modality probes to modular architectures that are biodegradable, immunologically tunable, and capable of incorporating multiple therapeutic and diagnostic functions in a programmable manner [214,215]. Parallel developments in precision nanomedicine underscore the importance of tailoring these carriers to patient-specific molecular profiles and treatment pathways rather than generic disease labels [216,217]. At the same time, artificial intelligence (AI), microfluidic manufacturing, and digital twin concepts are beginning to change the way nano-contrast systems are designed, optimized, and evaluated in silico before clinical deployment [218,219]. These developments extend and integrate the traditional platforms and theranostics paradigms developed in earlier sections.

### 7.1. Next-Generation Carrier Development

Next-generation nano-contrast carriers increasingly rely on biomimicry to meet the conflicting goals of high functional complexity, biocompatibility, and immune evasion. Cell membrane-coated nanoparticles, such as erythrocyte-, leukocyte-, or tumor cell membrane-coated inorganic or polymeric cores, have been shown to inherit native markers that extend circulation duration, modulate immune interactions, and facilitate homotypic or inflammatory targeting [220,221]. Similar strategies using platelet, stem cell, or hybrid membranes are being adapted to carriers bearing MRI, PET, and optical reporters to enable more selective delivery of theranostic payloads to vascular lesions or metastatic niches [222,223]. Exosome membrane-coated nanosystems introduce another level of biological sophistication, not only leveraging endogenous vesicle trafficking pathways but also using engineered cores to enable targeted imaging and therapy in oncology and neurology [224]. Together, these biomimetic carriers point to a trend toward living-like contrast agents that blend into existing physiological communication networks.

In parallel, porous and crystalline hybrid materials such as metal–organic frameworks (MOFs) are emerging as highly tunable scaffolds for multimodal imaging and combinatorial therapy. MOF-based nanoplatforms provide high surface areas, tunable pore chemistries, and, in many cases, intrinsic metal nodes that, in turn, can provide CT, MRI, or photoacoustic contrast, as well as coordinate chemotherapeutics, radiosensitizers, or immunomodulators [225]. Recent work focuses on biodegradable MOFs that dissociate into renally clearable components to address early concerns about the long-term persistence of inorganic frameworks [226]. Biomimetic MOFs that incorporate cell membranes or peptide ligands into their shells are a step toward targeting flexibility and can be designed for synergistic photothermal, photodynamic, and immunotherapies, in combination with multimodal imaging [227]. Similarly, emerging two-dimensional materials, such as MXenes, are garnering attention for their tunable biocompatibility, immunomodulatory properties, and capacity for the targeted delivery of bioactive agents [228].

Beyond new materials, next-generation carriers increasingly embody design principles focused on ultrasmall size, controlled degradability, and chemically defined architectures. Polymer drug conjugates exemplify this trend, in which drugs are covalently attached to biocompatible backbones, sometimes along with imaging moieties, enabling well-controlled drug-to-carrier ratios, release kinetics, and pharmacokinetics in nanotheranostics [229]. Emerging concepts in multifunctional nanomedicine promote the hierarchical, multicompartment structure in which imaging and therapeutic modules are in separate domains but are linked through programmable stimuli-responsive linkers [230]. Concurrently, the emergence of engineered nanobodies and other small targeting proteins is changing how carriers interact with molecular targets, with high-affinity recognition and enhanced tissue penetration and clearance compared with conventional antibodies [231]. Collectively, these strategies point toward carriers that are structurally simple enough to be easily manufactured at scale and cost-effectively, yet sophisticated enough to enable multimodality and multipayload theranostics.

### 7.2. Technology Integration and Digitalization

Digital technologies are rapidly permeating the design, optimization, and clinical deployment of nano-contrast carriers. On the discovery side, machine learning models based on physicochemical descriptors, pharmacokinetic profiles, and omics data are used to predict nano-bio interactions, toxicity, and therapeutic efficacy, thereby guiding the selection of carrier composition, size, and surface chemistry prior to synthesis [218,232]. Data-driven frameworks combining multidimensional characterization, preclinical outcomes, and clinical metadata are beginning to facilitate rational nanocarrier design and patient stratification for nanotherapies [233]. Complementary efforts in imaging science utilize AI for imaging optimization in dosing of contrast media, simulation of contrast enhancement, or even generation of virtual contrast images from nonenhanced scans, which raises the prospect for nano-contrast agents to be co-designed with AI pipelines that maximize the information content while minimizing administered dose [234,235].

Digitalization also applies to the interpretation and association of data from nano-contrast studies and the clinical decision-making process. Radiomics and AI-enhanced image analysis can extract hundreds of quantitative features from multimodal scans, which provide high-dimensional descriptors of tumor phenotype and microenvironment that complement molecular targeting by the carrier [236,237]. These imaging-derived features are increasingly integrated with genomic and transcriptomic data via AI pipelines to develop predictive models of response and toxicity that could be used to select patients most likely to benefit from specific nanotheranostic regimens [238]. At a systems level, medical digital twin concepts have been proposed in which continuously updated individualized virtual models couple imaging, clinical, and sensor data to simulate disease trajectories and therapy responses, providing a natural framework in which nano-contrast agents could serve as both probes and actuators in closed-loop precision medicine [219,239].

### 7.3. Precision Medicine Applications

Emerging applications of nano-contrast carriers are developing in the context of precision medicine, wherein the aim is to tailor therapeutic mechanisms and imaging readouts to the molecular and microenvironmental characteristics of an individual patient. Recent reviews of precision nanomedicine highlight the potential to develop nanocarriers matched to specific genomic changes, receptor profiles, or immune landscapes to enable more selective delivery of chemotherapeutics, gene editors, or immunomodulators, with inbuilt imaging capabilities for response tracking [216,240]. Another class of theranostic platforms that incorporates two functions (diagnostics and treatment) in a single construct is of special interest in the field of individualized oncology, as they allow real-time evaluation of intratumoral drug exposure, target engagement, and biological response [141,241]. Conceptually, nano-contrast agents thus evolve from static imaging adjuncts into dynamic companion tools that inform patient selection, dose adaptation, and therapy switching.

Theranostic paradigms in nuclear medicine provide a tangible model for integrating nano-contrast systems into precision workflows. In radiotheranostics, diagnostic and therapeutic radionuclides targeting the same molecular structure are used sequentially for disease staging, dosimetry estimation, and targeted radionuclide therapy, often with AI-assisted image analysis for more precise dose planning [242,243]. Transposing this logic to nano-contrast carriers implies platforms that could first be deployed at low doses for quantitative imaging and pharmacokinetic modeling, followed by therapeutic dosing in patients with favorable distribution profiles. Recent conceptual papers on personalized nanoparticles emphasize modular designs in which ligands, payloads, and imaging labels can be exchanged while maintaining a validated core scaffold, enabling rapid adaptation of the same platform across biomarker-defined subgroups [244,245].

Looking forward, precision applications of nano-contrast carriers will probably rely on the tight integration of advanced imaging, multi-omics profiling, and computational modeling. Multi-responsive, multimodal carriers can be used to report multiple microenvironmental parameters simultaneously (e.g., hypoxia, pH, or immune cell infiltration), thereby providing richer data streams for radiomics and AI models [225,246]. Digital twin frameworks propose how these agents could be assessed longitudinally using imaging data, which could then be fed into patient-specific models of change to test alternative treatment strategies in silico before clinical application [247]. At the same time, research on AI-guided nanomedicine emphasizes that achieving these visions will require standardized data infrastructures, interpretable models, and the early incorporation of regulatory and ethical considerations into design pipelines [218,248]. For multifunctional nano-contrast carriers, precision medicine therefore represents both opportunity and a demanding benchmark that will shape future innovation.

## 8. Conclusions

**Figure 10:**
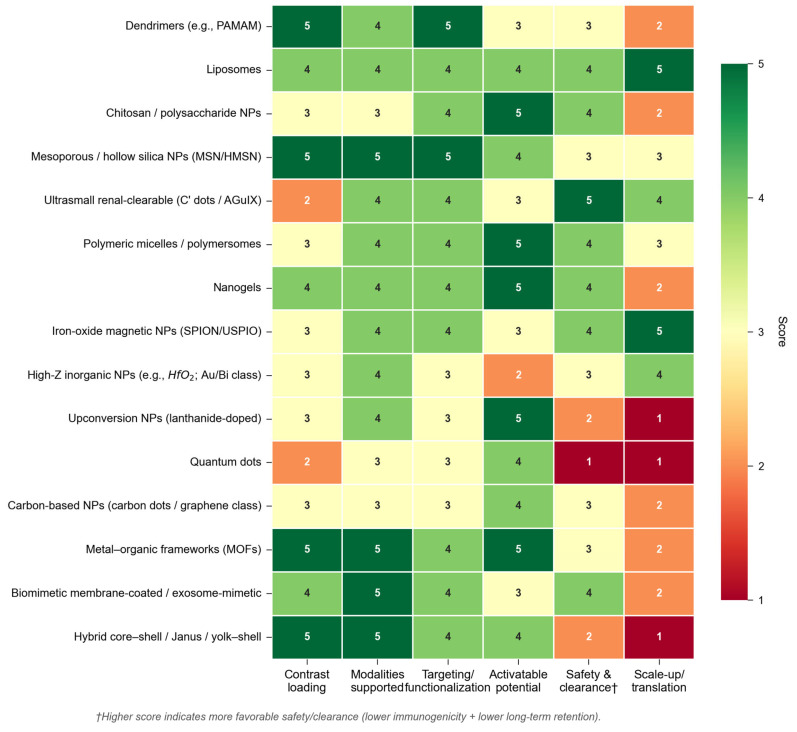
Heatmap scorecard comparing major nano-contrast carrier families across performance; scores range from 1 (least favorable) to 5 (most favorable). Scores were assigned by the authors based on the literature reviewed in this work.

Multifunctional nano-contrast agent carriers are now at a critical juncture between elegant materials science and the day-to-day demands of precision medicine, but bringing their design potential to fruition will require a deliberate rebalancing of complexity, predictability, and clinical utility across the entire design-translation continuum. Lessons from the dendrimer, liposome, chitosan, and silica platforms, as well as emerging biomimetic and hybrid systems, make it clear that high-density loading, multimodal readout, and stimuli-responsiveness can add considerably to the informational content and therapeutic leverage of molecular imaging, but they also teach us something quite important: even subtle changes in size, architecture, surface chemistry, or corona composition can have enormous effects on biodistribution, immune recognition, and long-term safety. These cross-platform trade-offs between functionality and real-world deployability are summarized in Figure 10.

A key research priority is therefore to advance from descriptive to mechanistic and, ultimately, predictive control of nano-bio interactions by tightly integrating advanced characterization, physiologically based pharmacokinetic and systems-biology modeling, high-content imaging, and rigorous immunotoxicology into carrier-optimization loops. In parallel, modular, hierarchically organized carrier architectures offer a tractable route toward standardization, scalable manufacturing, and lifecycle management, especially when combined with biodegradable or ultrasmall designs to support renal clearance and minimize chronic retention. At the translational interface, regulatory science needs to co-evolve with technology, with better guidance on acceptable complexity, quality attributes, and the data package for multifunctional theranostics, while developers have to be mindful of payer and workflow constraints, developing platforms that integrate seamlessly into existing imaging protocols, information systems, and treatment pathways. Finally, the most far-reaching opportunity is embedding nanocarrier contrast agents into AI-enabled multi-omics and digital twin frameworks, where rich, longitudinal, multiparametric imaging data are used not only to localize disease but also to dynamically update patient-specific models, refine risk stratification, and adapt treatment in real time. Pursuing this systems-level vision, rooted in mechanistic knowledge, manufacturability, safety, and clinical need, will determine whether multifunctional nano-contrast agents remain largely experimental demonstrations or evolve into indispensable, accessible tools for image-guided personalized therapy worldwide.

## Figures and Tables

**Table 1:** Traditional nanocarrier platforms are used as contrast-agent carriers in molecular imaging.

Platform	Typical Particle Size (nm)	Payload Incorporation Strategies	Modalities Commonly Supported	Advantages	Limitations	Representative Exemplar(s)	Refs.
Dendrimers (e.g., PAMAM)	~2–15 (generation-dependent)	- Multivalent conjugation of chelated metals (e.g., Gd) - Attachment of fluorophores/radiometals - High-Z element incorporation	MRI; optical; PET/SPECT; CT (via high-Z loading)	- Precise, nearly monodisperse architectures - High payload capacity and controlled stoichiometry	- Synthetic complexity and cost - Potential immune interactions (higher generations) - Need for robust clearance design	- Gadomer-17 (MR angiography; clinical) - Dendrimer CT designs (preclinical)	[24,25,26,27]
Liposomes (phospholipid vesicles; often PEGylated)	~80–200 (commonly ~100)	- Hydrophilic agents in aqueous core - Amphiphilic probes in bilayer - Surface ligand targeting - Multimodal co-loading	MRI; CT; optical; PET/SPECT	- Clinically mature (regulatory familiarity) - Flexible loading (core vs. bilayer) - Scalable theranostic platform	- Leakage/instability and batch control issues - MPS uptake without optimized surfaces - Anti-PEG immunogenicity risks	- Gd-labelled nanoliposomes (preclinical) - Broad liposome probes across modalities	[28,29,30,31]
Chitosan-based nanoparticles (polymeric matrix or coating)	~50–300 (often sub-micron, formulation-dependent)	- Matrix encapsulation of dyes/chelates - Coating for inorganic cores (e.g., magnetic) - Ligand functionalization	MRI (often via magnetic cores); optical/fluorescence; CT (via hybrid designs)	- Biocompatible, cationic, mucoadhesive - Abundant groups for electrostatic loading - Excellent stabilizing shell	- pH/ionic-strength dependent solubility - Batch variability (ionotropic gelation) - Limited clinical translation	- Cancer theranostic nanosystems (reviewed) - Ionotropic gelation formulations	[9,32,33,34]
Silica nanoparticles (MSNs/HMSNs; ultrasmall silica variants)	MSNs typically ~50–200; ultrasmall silica ~6–10	- Pore-based loading (adsorption/entrapment) - Doping with metals (e.g., Gd) and fluorophores - Modular core–shell/hollow architectures	MRI; CT; optical; PET/SPECT; (optionally) US in hybrid designs	- High surface area and tunable pores - Robust framework chemistry - Segregated co-loading capabilities	- Macrophage uptake and retention risks - Design-dependent biodegradation - Scale-up and QC challenges	- Cornell dots/C’Dots (clinical trials/IND) - MSNs (broad clinical evaluation)	[35,36,37,38]

Abbreviations: CT—computed tomography; Gd—gadolinium; HMSN—hollow mesoporous silica nanoparticle; IND—investigational new drug; MR angiography; MRI—magnetic resonance imaging; MSN—mesoporous silica nanoparticle; MPS—mononuclear phagocyte system; PAMAM—poly(amidoamine); PEG—polyethylene glycol; PET—positron emission tomography; SPECT—single-photon emission computed tomography; US—ultrasound.

**Table 2:** Engineering toolbox matrix linking design levers to biological consequences and downstream imaging effects.

Design Strategy	Example Implementations	Biological Consequences	Imaging Effects	Trade-Off/Failure Mode	Refs.
Corona control/biological identity engineering	- Pre-conditioning in biofluids - Artificial protein coronas - Corona-resistant surfaces	- Alters opsonization and immune recognition - Modulates cell–surface interactions	- Modulates non-specific background and targeting fidelity - Improves in vivo reproducibility	- In vivo corona exchange - Batch-to-batch variability - Masking of functional ligands	[71,72,73,74,75]
PEGylation (stealth polymer)	- PEG brushes on NPs - PEG–lipids for liposomes	- Reduces protein adsorption; prolongs circulation - Risk of anti-PEG immune responses and ABC	- Extends initial imaging window and blood-pool suppression - Reduced reproducibility upon repeat dosing (ABC)	- ABC phenomenon and hypersensitivity - Reduced cellular uptake - Efficacy loss on re-administration	[76,77,78,79]
Zwitterionic/ultra-hydrophilic antifouling coatings	- Carboxybetaine/sulfobetaine polymers - Phosphorylcholine (MPC) coatings	- Suppresses non-specific protein adsorption - Reduces coagulation/complement activation	- Lowers off-target background - Stabilizes biodistribution for quantitative interpretation	- May reduce receptor-mediated uptake - Synthetic/scale-up complexity - Grafting stability under shear	[80,81,82]
Biomimetic cloaking	- Membrane-coated NPs (RBC, platelet, cancer cell) - Hybrid membrane coatings	- Adds “self” cues to reduce MPS clearance - Enables homotypic/tumor tropism	- Increases target-to-background ratio via prolonged circulation - Improves disease-site accumulation	- Source variability and membrane integrity issues - Protein orientation challenges - Biosafety/sterility burden	[83,84,85]
Size regime/clearance pathway design	- Ultrasmall (renal-clearable) designs - Size/shape optimization	- Shifts clearance route (renal vs. hepatic/MPS) - Alters tissue penetration and sequestration	- Renal-clearable: faster washout, cleaner whole-body imaging - Larger: higher payload but increased liver/spleen background	- Fast clearance reduces target accumulation - Size-dependent loss of material properties - Off-target organ retention.	[86,87,88,89]
Targeting ligand class & presentation	- Peptides (e.g., RGD), antibodies, aptamers - Oriented conjugation chemistries	- Enables receptor-mediated binding/internalization - Susceptible to ligand screening by adsorbed coronas	- Higher specificity/contrast if binding is preserved - Supports molecular imaging beyond passive accumulation	- Receptor heterogeneity and ligand immunogenicity - Corona masking of ligands - Limited overall tumor delivery efficiency	[90,91,92]
Ligand density/multivalency/spatial organization	- Controlled densities (clustered vs. dispersed) - Super-selective multivalent designs	- Tunes avidity, receptor clustering, and internalization - Shifts cell-type selectivity via threshold effects	- Improves sensitivity at low receptor expression - Sharpens discrimination (lesion-to-background contrast)	- Over-dense ligands increase non-specific opsonization - Receptor saturation - Altered PK	[93,94]
Stimuli-responsive surface transformations	- pH/redox/enzyme-cleavable shells - Charge-switching/sheddable stealth layers	- Switches from stealth to interactive in TME/endosomes - Enhances endosomal escape/intracellular delivery	- Activatable accumulation and improved intracellular signal - Reduces off-target interactions	- Premature activation in circulation - Heterogeneous activation across lesions - Incomplete switching	[95,96,97]
Enzyme-activatable reporting (activity-based imaging)	- Protease-activated fluorogenic probes - Activatable cell-penetrating peptides	- Reports local enzymatic activity (not just NP presence) - Increases functional specificity	- High contrast via signal “turn-on” at active sites - Maps pathology-related enzyme activity	- Activated signal diffusion - Off-target enzyme activity - Substrate specificity constraints	[98,99]
Multimodal + nuclear-labeling for quantitation	- PET/SPECT radiolabeling of NPs - MRI–PET or MRI–optical composites	- Minimally alters biology if labeling is stable - Enables rigorous PK/biodistribution validation	- Quantitative whole-body tracking (PET/SPECT) + anatomical co-registration - Strengthens interpretability	- Radiolabel instability misreports biodistribution - Signal decoupling (different sensitivities/time scales) - High formulation complexity	[100,101,102]

Abbreviations: ABC—accelerated blood clearance; MPC—2-methacryloyloxyethyl phosphorylcholine; MPS—mononuclear phagocyte system; MRI—magnetic resonance imaging; NP—nanoparticle; PEG—polyethylene glycol; PET—positron emission tomography; PK—pharmacokinetics; RBC—red blood cell; RGD—Arg–Gly–Asp; SPECT—single-photon emission computed tomography; TME—tumor microenvironment.

**Table 3:** Multimodal imaging combinations in nano-contrast systems.

Modality Combination	Typical Integration Approach	Complementary Value	Representative System(s)	Key Limitations/Translation Considerations	Refs.
PET/MRI	- Radiometal chelation or chelator-free incorporation - Magnetic core provides MR contrast	- PET: High sensitivity & whole-body quantitation - MRI: Soft-tissue anatomy & functional readouts	^64^Cu-integrated iron oxide core–shell NPs; radiolabeled SPION constructs	- Radiolabel stability & complex QC - PET/MR attenuation correction artifacts - Dose/synthesis logistics	[154,155,156]
SPECT/MRI	- 99mTc/111In labeling via chelators on SPIONs - Optional targeting ligands	- SPECT: Accessible nuclear quantitation - MRI: Anatomical localization & vascular readouts	SPION-based SPECT/MRI tumor probes	- Lower sensitivity/resolution than PET - Radiolabeling reproducibility - MR susceptibility blooming (biases lesion sizing)	[154,156,157]
PET/CT	- High-Z (e.g., Au) nanoplatforms - PET radionuclide labeling (DOTA/NOTA)	- PET: Molecular sensitivity & quantitation - CT: Fast anatomical context & additional contrast	^68^Ga-labeled dendrimer-entrapped AuNPs (PET/CT theranostic platform)	- CT radiation dose - Limited soft-tissue contrast - Complex regulatory pathways	[158]
CT/MRI	- Hybrid high-Z + magnetic nanohybrids (e.g., Bi–Fe oxide)	- CT: Electron-density (HU) mapping - MRI: Soft-tissue detail & multiparametric sequences	Dextran-coated bismuth–iron oxide nanohybrids (CT + T_2_ MRI)	- CT beam-hardening artifacts - MR susceptibility issues - Long-term clearance of dense cores	[159]
PET/Optical (fluorescence/NIRF)	- Fluorophore embedded/linked to NPs - PET radionuclide labeling (124I or radiometal)	- PET: Whole-body quantitation - Optical: High-resolution superficial/intraoperative guidance	Ultrasmall silica C-dot PET–optical probe translated clinically	- Optical depth limitations - Fluorescence quenching/photobleaching - Matching PK across dual reporters	[16,154]
MRI/Fluorescence	- Magneto-fluorescent assemblies (SPION + dye/QD) - Covalent linking, encapsulation, or core–satellite designs	- MRI: Deep-tissue localization - Fluorescence: Cell-level microscopy & multiplexing	Multicore magneto-fluorescent nanoassemblies (reviewed design archetypes)	- Fluorescence quenching near magnetic cores - Dye leakage - MR sensitivity limits for sparse targets	[160]
CT/Photoacoustic (PA)	- Plasmonic absorbers (e.g., Au-based) tuned for NIR - High-Z content for CT	- CT: Anatomy & HU quantitation - PA: High-contrast optical absorption maps at depth	Thin-layer–protected AuNPs for PA/CT imaging	- Laser fluence limits & heating risks - PA signal dependence on local optical fluence - CT radiation dose	[161]
MRI/Photoacoustic (PA)	- NIR-absorbing nanomaterials - MR-active doping (e.g., Mn) or composite designs	- MRI: Deep anatomical/functional context - PA: Sensitive absorption contrast (vascular/tumor interfaces)	Mn-doped Prussian blue nanoparticles for MRI/PA (and often photothermal capability)	- Potential metal-ion release - PA fluence heterogeneity (complicates quantitation) - Multi-parameter QC burden	[162]
Tri-modal (e.g., PET/MRI/Optical)	- Co-packaging of radionuclide + MR-active core + fluorophore	- Cross-validates biodistribution/targeting - Supports whole-body quantitation + high-resolution local readouts	Tri-modal probe archetypes summarized across PET/SPECT–MR–optical designs	- High synthetic complexity - Harder CMC/QA and regulatory review - Signal decoupling if labels dissociate	[154]

Abbreviations: AuNP—gold nanoparticle; CMC—chemistry, manufacturing, and controls; CT—computed tomography; DOTA—1,4,7,10-tetraazacyclododecane-1,4,7,10-tetraacetic acid; HU—Hounsfield unit; MR—magnetic resonance; MRI—magnetic resonance imaging; NIR—near-infrared; NIRF—near-infrared fluorescence; NOTA—1,4,7-triazacyclononane-1,4,7-triacetic acid; NP—nanoparticle; PA—photoacoustic; PET—positron emission tomography; PK—pharmacokinetics; QA—quality assurance; QC—quality control; QD—quantum dot; SPION—superparamagnetic iron oxide nanoparticle; SPECT—single-photon emission computed tomography.

**Table 4:** Clinical translation status of representative nano-contrast and theranostic platforms.

Platform (Exemplar)	Nanomaterial Class/Active Component	Primary Clinical Role	Imaging Modality(ies) Used Clinically	Most Advanced Translation Status	NCT Identifier(s) (Representative)	Key Translational Constraints
Ferumoxytol (Ferabright™; related ferumoxytol products)	USPIO iron oxide suspension	Brain tumor MRI (lesions with disrupted BBB); broader FE-MRI applications in vasculature/inflammation (off-label literature)	MRI (T1/T2*/susceptibility-based)	FDA-approved for brain MRI indication (October 2025)	NCT00659126; NCT02452216	- Monitored IV administration (hypersensitivity risk) - Protocol-dependent susceptibility effects - Timing/dose optimization required
Ferumoxides (Feridex^®^/Endorem^®^)	SPIO iron oxide formulation	Liver lesion detection via RES/Kupffer uptake	MRI (predominantly T2/T2*)	Historically approved; discontinued/withdrawn in many markets	-(legacy approvals; trials often pre-registry era)	- Discontinued/limited access - Strong susceptibility reduces lesion conspicuity - Complicates scanner standardization
Ferucarbotran (Resovist^®^/Cliavist^®^)	SPIO iron oxide formulation	Liver MRI (RES imaging)	MRI (T2/T2*)	Historically approved; reduced availability/withdrawn	-(legacy approvals; trials often pre-registry era)	- Market discontinuity and heterogeneous adoption - Susceptibility artifacts and workflow dependence
Ferumoxtran-10 (Combidex^®^/Sinerem^®^; “Ferrotran^®^”)	USPIO iron oxide nanoparticle (lymphotropic)	Nodal staging (e.g., pelvic/prostate lymph nodes)	MRI	EMA withdrawn (2007); renewed development (Ferrotran) in late-stage trials	NCT04261777; NCT02751606	- Rigorous reader training/standardized interpretation required - Susceptibility appearance varies with timing/sequence
AGuIX^®^	Ultrasmall polysiloxane matrix bearing Gd chelates	MRI-visible radiosensitizer used with RT (brain metastases; GBM)	MRI (alongside RT workflows)	Early clinical evaluation (NANORAD, NANO-GBM trials)	NCT02820454; NCT04881032	- Consistent tumor accumulation/dosimetry needed - Integration into RT planning - Long-term metal retention and PK follow-up
Cornell dots (^124^I-cRGDY–PEG–C dots)	Ultrasmall silica-based, renal-clearable nanoparticle; radiolabeled + fluorescent	Targeted PET–optical imaging (e.g., melanoma)	PET/CT + NIR fluorescence	First-in-human/microdosing studies (tumor targeting, dosimetry)	NCT01266096	- Microdose/limited payload constrains signal - Requires robust radiochemistry, QC, and scalable GMP
NBTXR3 (Hensify^®^)	High-Z hafnium oxide nanoparticles	Intratumoral radioenhancer (theranostic-by-design via imageable high-Z depot + RT enhancement)	CT (procedure-dependent) + RT	CE marking (Act.In.Sarc); ongoing global trials	NCT02379845	- Accurate intratumoral delivery and homogeneous distribution required - Depends on tumor accessibility and injection workflow

Abbreviations: AGuIX—ultrasmall polysiloxane gadolinium nanoparticle platform; BBB—blood–brain barrier; CE—Conformité Européenne; CT—computed tomography; EMA—European Medicines Agency; FDA—Food and Drug Administration; FE-MRI—ferumoxytol-enhanced MRI; GBM—glioblastoma; Gd—gadolinium; GMP—Good Manufacturing Practice; IV—intravenous; MRI—magnetic resonance imaging; NCT—ClinicalTrials.gov identifier; NIR—near-infrared; PEG—polyethylene glycol; PET—positron emission tomography; PK—pharmacokinetics; QC—quality control; RES—reticuloendothelial system; RT—radiotherapy; SPIO—superparamagnetic iron oxide; USPIO—ultrasmall superparamagnetic iron oxide.

## Data Availability

No new data were created or analyzed in this study. Data sharing is not applicable to this article.

## Abbreviations

The following abbreviations are used in this manuscript:

^124^I	Iodine-124 (radionuclide used for PET labeling)
ADME	Absorption, distribution, metabolism, and excretion
AGuIX^®^	Ultrasmall polysiloxane gadolinium-based nanoparticle platform used as an MRI-visible radiosensitizer/contrast agent
AI	Artificial intelligence
C dots	Cornell dots: ultrasmall silica-based optical/PET imaging nanoparticle probe
cRGDY	Cyclic Arg–Gly–Asp–Tyr peptide (integrin-targeting RGD variant)
CT	Computed tomography
DELIVER	A translational framework/guideline for bringing nanomedicines to the clinic
DNA	Deoxyribonucleic acid
DTPA	Diethylenetriaminepentaacetic acid (metal chelator)
EPR	Enhanced permeability and retention (effect)
Fe^3+^	Iron(III) ion
FDA	US Food and Drug Administration
FE-MRI	Ferumoxytol-enhanced MRI
GBCA(s)	Gadolinium-based contrast agent(s)
GBM	Glioblastoma (often glioblastoma multiforme)
Gd	Gadolinium
Gd(III)	Gadolinium(III) ion
HMSN(s)	Hollow mesoporous silica nanoparticle(s)
M1	Pro-inflammatory (classically activated) macrophage phenotype
M2	Immunosuppressive/tissue-repair (alternatively activated) macrophage phenotype
Mn^2+^	Manganese(II) ion
MOF(s)	Metal–organic framework(s)
MR	Magnetic resonance
MR/US	Magnetic resonance/ultrasound (combined imaging)
MRI	Magnetic resonance imaging
MPS	Mononuclear phagocyte system
MSN(s)	Mesoporous silica nanoparticle(s)
MSNs–Pt	Platinum-embedded/decorated mesoporous silica nanoparticles
NANO-GBM	Clinical trial of AGuIX nanoparticles with chemoradiation in glioblastoma
NANORAD	Phase I study protocol using gadolinium nanoparticles with radiotherapy (brain metastases)
PAMAM	Poly(amidoamine) (dendrimer)
PBPK	Physiologically based pharmacokinetic (modeling/framework)
PEG	Polyethylene glycol
PE-DTPA	Phosphoethanolamine–DTPA (a Gd-chelating lipid conjugate)
PET	Positron emission tomography
PET/CT	Hybrid positron emission tomography/computed tomography
PET/MR (PET/MRI)	Hybrid positron emission tomography/magnetic resonance (imaging)
RGD	Arg–Gly–Asp peptide motif (integrin-binding)
ROS	Reactive oxygen species
r_1_	Longitudinal relaxivity (T_1_ relaxivity)
SPECT	Single-photon emission computed tomography
SPECT/CT	Hybrid single-photon emission CT/computed tomography
SPECT/MR (SPECT/MRI)	Hybrid single-photon emission CT/magnetic resonance (imaging)
T_1_	Longitudinal relaxation time (T_1_)/T_1_-weighted contrast
T_2_	Transverse relaxation time (T_2_)/T_2_-weighted contrast
US	Ultrasound
